# The Role of Coupled Positive Feedback in the Expression of the SPI1 Type Three Secretion System in *Salmonella*

**DOI:** 10.1371/journal.ppat.1001025

**Published:** 2010-07-29

**Authors:** Supreet Saini, Jeremy R. Ellermeier, James M. Slauch, Christopher V. Rao

**Affiliations:** 1 Department of Chemical and Biomolecular Engineering, University of Illinois at Urbana-Champaign, Urbana, Illinois, United States of America; 2 Department of Microbiology, University of Illinois at Urbana-Champaign, Urbana, Illinois, United States of America; 3 College of Medicine, University of Illinois at Urbana-Champaign, Urbana, Illinois, United States of America; Washington University, United States of America

## Abstract

*Salmonella enterica* serovar Typhimurium is a common food-borne pathogen that induces inflammatory diarrhea and invades intestinal epithelial cells using a type three secretion system (T3SS) encoded within *Salmonella* pathogenicity island 1 (SPI1). The genes encoding the SPI1 T3SS are tightly regulated by a network of interacting transcriptional regulators involving three coupled positive feedback loops. While the core architecture of the SPI1 gene circuit has been determined, the relative roles of these interacting regulators and associated feedback loops are still unknown. To determine the function of this circuit, we measured gene expression dynamics at both population and single-cell resolution in a number of SPI1 regulatory mutants. Using these data, we constructed a mathematical model of the SPI1 gene circuit. Analysis of the model predicted that the circuit serves two functions. The first is to place a threshold on SPI1 activation, ensuring that the genes encoding the T3SS are expressed only in response to the appropriate combination of environmental and cellular cues. The second is to amplify SPI1 gene expression. To experimentally test these predictions, we rewired the SPI1 genetic circuit by changing its regulatory architecture. This enabled us to directly test our predictions regarding the function of the circuit by varying the strength and dynamics of the activating signal. Collectively, our experimental and computational results enable us to deconstruct this complex circuit and determine the role of its individual components in regulating SPI1 gene expression dynamics.

## Author Summary

*Salmonella* is a causative agent for a wide range of diseases in humans, including gastroenteritis and enteric fever. A key step in the infection process occurs when *Salmonella* invades intestinal epithelial cells using a molecular hypodermic needle. *Salmonella* uses these needles to inject proteins into host cells that enable the bacterium to enter and replicate within them. The production of these needles, and the corollary decision to invade the host, is tightly controlled by a complex network of interacting regulatory proteins that, when studied individually, seemingly have either redundant or antagonizing effects. To understand how this ensemble of regulators dynamically controls the expression of these invasion genes, we systematically deconstructed the network and then used this information to analyze their composite behavior by computer simulation. Our analysis demonstrates that this regulatory network ensures that the invasion genes are expressed only when the invasion signals, a combination of environmental and cellular cues, exceed a defined threshold. Once induced, this network further amplifies and accelerates the expression of the invasion genes. These results further our understanding of this important pathogen by unraveling a key mechanism during infection, namely the decision to invade.

## Introduction

*Salmonella enterica* is a common food-borne pathogen that causes an array of diseases in humans, ranging from self-limiting gastroenteritis to life-threatening systemic infections [1], [2]. The bacterium initiates infection by invading intestinal epithelial cells using a type three secretion system (T3SS) encoded within a forty kilobase region of the chromosome called *Salmonella* Pathogenicity Island 1 (SPI1) [3], [4], [5], [6], [7], [8]. The bacterium uses this T3SS to inject proteins into the cytoplasm of host cells [9], [10], [11]. The injected proteins commandeer the host cell actin-cytoskeleton machinery and promote the uptake of the bacterium into these otherwise non-phagocytic cells [12], [13], [14], [15]. The genes encoding the SPI1 T3SS are tightly regulated by a network of interacting transcriptional regulators that are responsive to a combination of environmental and intracellular signals [16], [17], [18]. These signals are presumably used by *Salmonella* as anatomical cues for initiating invasion and also for coordinating SPI1 gene expression with other cellular processes, most notably adhesion and motility [19], [20], [21], [22], [23], [24].

**Figure 1:**
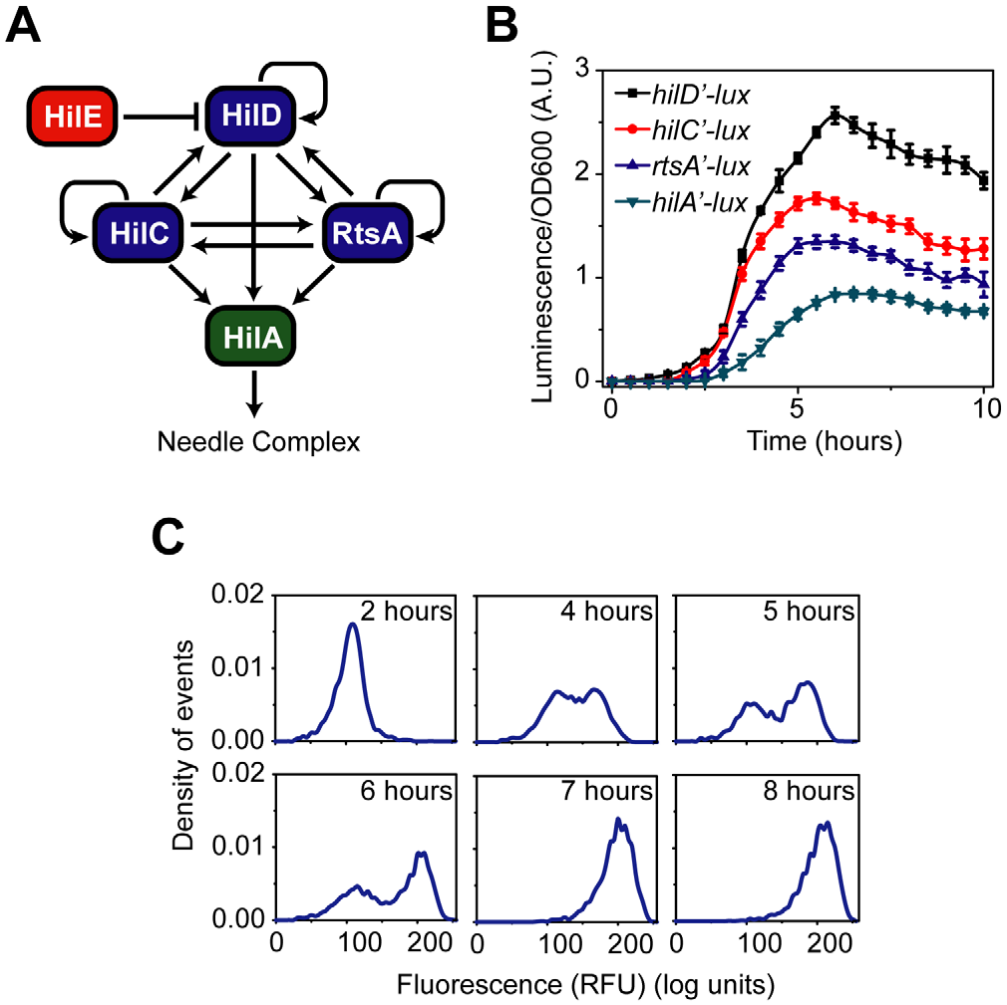
SPI1 gene expression is hierarchical and exhibits a switch-like transition from the “off” to the “on” state. (A) Diagram of the SPI1 gene circuit. HilA is the master SPI1 regulator as it activates the expression of the genes encoding the T3SS. HilA, in turn, is regulated by HilC, HilD, and RtsA. These three regulators can independently activate HilA expression. They can also activate their own expression and that of each other's. HilE represses the activity of HilD by binding to it and preventing it from activating its target promoters. (B) Time-course dynamics of P*_hilD_* (pSS074), P*_hilC_* (pSS075), P*_rtsA_* (pSS076), and P*_hilA_* (pSS077) promoter activities in wild-type cells as determined using luciferase transcriptional reporters. To induce SPI1 gene expression, cells were first grown overnight in LB/no salt and then sub-cultured into fresh LB/1% NaCl conditions to an OD of 0.05 and grown statically. Luminescence values were normalized with the OD_600_ absorbance to account for cell density. Average promoter activities from three independent experiments on separate days are reported. For each experiment, six samples were tested. Error-bars indicate standard deviation. (C) Dynamics of P*_hilA_* (pSS055) promoter activity in wild-type cells as determined using green fluorescent protein (GFP) transcriptional fusions and flow cytometry. The SPI1 gene expression was induced as described above. Samples were collected at the indicated times and arrested in their respective state by adding chloramphenicol. Approximately 30,000 cell measurements were used to construct each histogram. As a control, we expressed GFP from a constitutive promoter and observed continuous, rheostatic-like expression dynamics and a homogenous response in the population (**Figure S1E**). Strain genotypes and plasmid descriptions are provided in **Tables 1** and **2**.

The master regulator for the SPI1 gene circuit is HilA, a transcription factor that contains a DNA-binding motif belonging to the OmpR/ToxR family [4] and a large C-terminal domain of unknown function [25]. HilA activates the expression of the genes encoding the structural components of the SPI1 T3SS [4], [26], [27], [28]. HilA also activates the expression of an AraC-like transcription factor, InvF, involved in regulating the expression of the SPI1 secreted effector proteins and their cognate chaperones [29], [30]. HilA expression, in turn, is regulated by three AraC-like transcription factors - HilC, HilD, and RtsA – with homologous DNA binding domains [22], [31], [32]. Both *hilC* and *hilD* are encoded within SPI1 whereas *rtsA* is encoded elsewhere on the chromosome. These three transcription factors can independently activate HilA expression. They can also activate each others' and their own expression [16]. Specifically, HilC, HilD, and RtsA are all capable of individually activating the P*_hilA_* P*_hilC_*, P*_hilD_*, and P*_rtsA_* promoters. These auto-regulatory interactions result in three coupled positive feedback loops comprising HilC, HilD, and RtsA, the output of each capable of activating HilA expression (**Figure 1A**). Of the three, HilD is dominant, as there is no HilA expression in its absence [33]. This reflects the fact that many activating signals, both environmental and intracellular, affect SPI1 gene expression by modifying the activity of HilD protein [16], [18], [19], [23], [34], [35]. In addition to positive regulation, SPI1 gene expression is also subject to negative regulation. HilE, a protein of unknown structure encoded outside SPI1, binds HilD [34] and prevents it from activating its target promoters.

While the core architecture of the SPI1 gene circuit has been determined (**Figure 1A**), the functions of these interacting regulators and associated feedback loops are still unknown. Therefore, to deconstruct this circuit, we measured gene expression dynamics at both population and single-cell resolution in a number of SPI1 regulatory mutants. Based on these experimental results, we constructed a simple mathematical model of the SPI1 gene circuit. Using the model, we demonstrate that the circuit serves two functions. The first is to place a threshold on SPI1 activation, ensuring that the genes encoding the T3SS are expressed only in response to the appropriate combination of environmental and cellular cues. The second is to amplify SPI1 gene expression. To experimentally test these two predictions, we rewired the SPI1 network by changing its regulatory architecture. The resulting experimental and computational analyses underpin an integrated model for the regulation of SPI1 gene expression.

## Results

### Dynamics of SPI1 gene expression

To investigate the dynamics of SPI1 gene expression, we grew cells statically in Luria-Bertani (LB) medium using 1% NaCl as the inducing signal. Growth in low-oxygen and high-salt conditions has previously been shown to induce SPI1 gene expression *in vitro* [4], [28]. In these experiments, we grew the cells overnight in LB/no salt and then sub-cultured them into fresh LB/1% NaCl medium, thus inducing a transition from SPI1-repressing to SPI1-inducing conditions. We employed two different reporter systems to measure gene expression. In our bulk, population-level experiments, we measured gene expression using plasmid-based promoter fusions to the luciferase operon, *luxCDABE*, from *Photorhabdus luminescens* [36], [37]. In our single-cell experiments, we employed promoter fusions to the green fluorescent protein (GFP) using an otherwise identical plasmid-based system [38].

The advantage of using the luciferase reporter system is that it is sensitive to dynamic changes in promoter activity, particularly at low levels of expression [39]. However, bacterial luciferase produces insufficient light for single-cell studies, hence the need for fluorescent reporters. We also note that the bacterial luciferase reporter system imposes a metabolic burden due to the production of the luciferase substrate, tetradecanal, by LuxC, LuxD, and LuxE [40]. To account for any potential biases associated with bacterial luciferase, we repeated a number of population-level experiments using the GFP reporters with similar results (results not shown).

We measured gene expression dynamics in wild-type cells using the luciferase reporter system. After a brief lag following subculture, we found that the P*_hilD_* and P*_hilA_* promoters were activated in a sequential manner, consistent with HilD being necessary for HilA expression (**Figure 1B**). In the case of the P*_hilC_* and P*_rtsA_* promoters, we found that they were activated at roughly the same time as the P*_hilD_* promoter. This hierarchy can also be seen when the expression values are normalized with respect to their maximal value (**Figure S1A**). These results indicate that there is a temporal hierarchy in SPI1 gene expression, with HilC, HilD, and RtsA at the top of the transcriptional cascade and HilA at the bottom. A similar hierarchy has also been observed in the activation of the downstream promoters regulating the expression of the genes encoding the T3SS and secreted effector proteins [41].

We also measured wild-type gene expression dynamics using flow cytometry in order to determine how individual cells within the population behave during SPI1 induction. In the case of the P*_hilA_* promoter, the dynamics were not continuous; rather, individual cells transitioned from an “off” to the “on” state in a switch-like manner (**Figure 1C**). By switch-like, we mean that the individual cells exist in one of two expression states. At intermediate times, transient heterogeneity in the population is observed, with most cells existing in either the “off” or “on” state. Similar switch-like dynamics were also observed for the P*_hilC_*, P*_hilD_*, and P*_rtsA_* promoters, with a comparable hierarchy in activation times as observed in the population data (**Figure S1B–D**). We note that heterogeneity in SPI1 gene expression has been previously observed by others [42]. As the SPI1 gene circuit involves multiple interacting positive feedback loops, these results are not surprising. In particular, positive feedback is known to be an integral element in many cellular switches [43]. To identify the genesis of this behavior, we further investigated the regulation of SPI1 gene expression.

### Induction of the SPI1 gene circuit begins with a step increase in P*_hilD_* promoter activity

**Figure 2:**
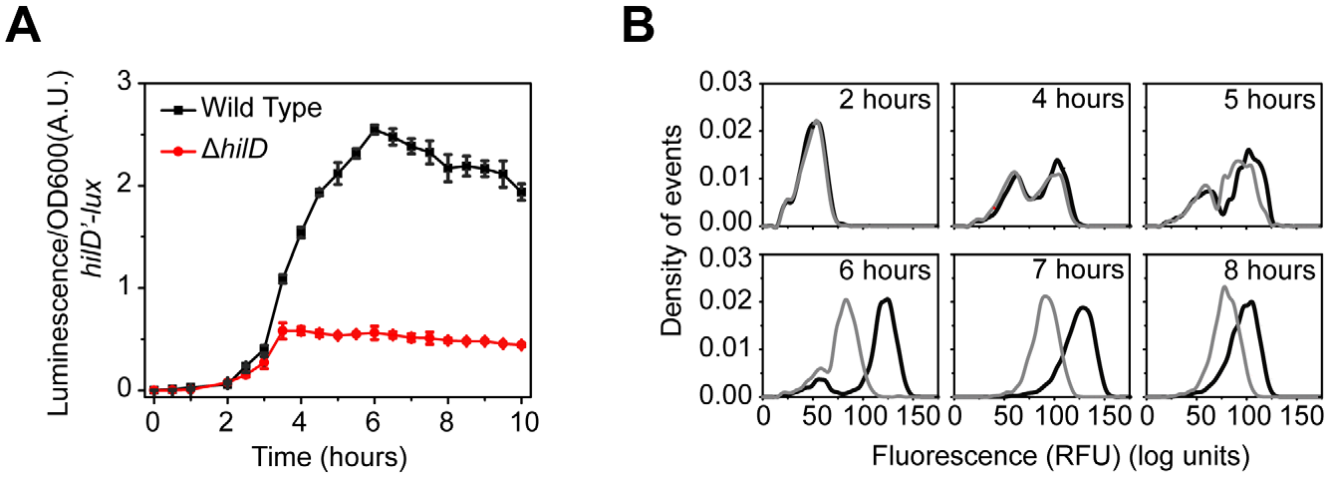
SPI1 gene expression is induced by a step increase in P*_hilD_* promoter activity. (A) Comparison of time-course dynamics for P*_hilD_* (pSS074) promoter activities in wild type (black) and a Δ*hilD* mutant (JS253, red) as determined using luciferase transcriptional reporters. (B) Comparison of P*_hilD_* (pSS072) promoter activity in wild type (black) and a Δ*hilD* mutant (JS253, grey) as determined using GFP transcriptional reporters and flow cytometry. Note that the activation of the P*_hilD_* promoter is switch-like both in wild type and the Δ*hilD* mutant. Experiments were performed as described in **Figure 1**.

HilD is necessary for HilA expression. Even though HilC and RtsA can independently activate HilA expression when constitutively expressed from ectopic promoters, these two regulators are incapable of doing so in the absence of HilD when expressed from their native promoters [16]. Therefore, to understand the role of HilD, we measured gene expression dynamics in a Δ*hilD* mutant using the luciferase reporters. In the case of the P*_hilA_* promoter, we observed no activity in the absence of HilD (data not shown), consistent with previous reports [16], [33]. In the case of the P*_hilD_* promoter, we observed a weak, step-like increase in activity in the absence of HilD (**Figure 2A**). When we performed identical experiments using flow cytometry, we found that the P*_hilD_* promoter again transitions from an “off” to the “on” state in a switch-like manner (**Figure 2B**). These results are identical to what is observed in wild-type cells, the only difference being that the magnitude of expression is significantly reduced when HilD is not present. We also performed identical experiments in a ΔSPI1 Δ*rtsA* mutant and observed the same response (**Figure S2A**), indicating that the transient switch in P*_hilD_* promoter activity is not due to any SPI1 regulator but rather factors external to SPI1.

These results demonstrate that the SPI1 gene circuit is activated by a step increase in P*_hilD_* promoter activity. This signal is then amplified by a positive feedback loop involving HilD. As discussed below, HilC and RtsA serve to further amplify this signal. Interestingly, the heterogeneity in SPI1 activation is not due to the interacting positive feedback loops within the circuit but rather is intrinsic to the activating signal. The signals activating the P*_hilD_* promoter, however, are unknown. While multiple global regulators are known to affect SPI1 gene expression [44], these regulators appear to affect the activity of the HilD protein and not its promoter [16], [18], [19], [23], [35].

With regards to HilC and RtsA, we found that the P*_hilC_* promoter was active in absence of HilD, though at a reduced level, whereas the P*_rtsA_* promoter was effectively off (**Figure S2B**). However, even though the P*_hilC_* promoter is active in the absence of HilD, HilA is not expressed. These results suggest that activation of the P*_hilD_* promoter is the trigger mechanism for induction of SPI1 gene expression. Interestingly, when we assayed P*_hilC_* promoter activity in a Δ*hilD* mutant using flow cytometry, we found that the dynamics were not switch-like but rather continuous and rheostatic (**Figure S2C**). This homogeneity within the population indicates that the signal activating the P*_hilC_* promoter is fundamentally different than the one activating the P*_hilD_* promoter.

### HilC and RtsA function as transcriptional amplifiers and accelerators

**Figure 3:**
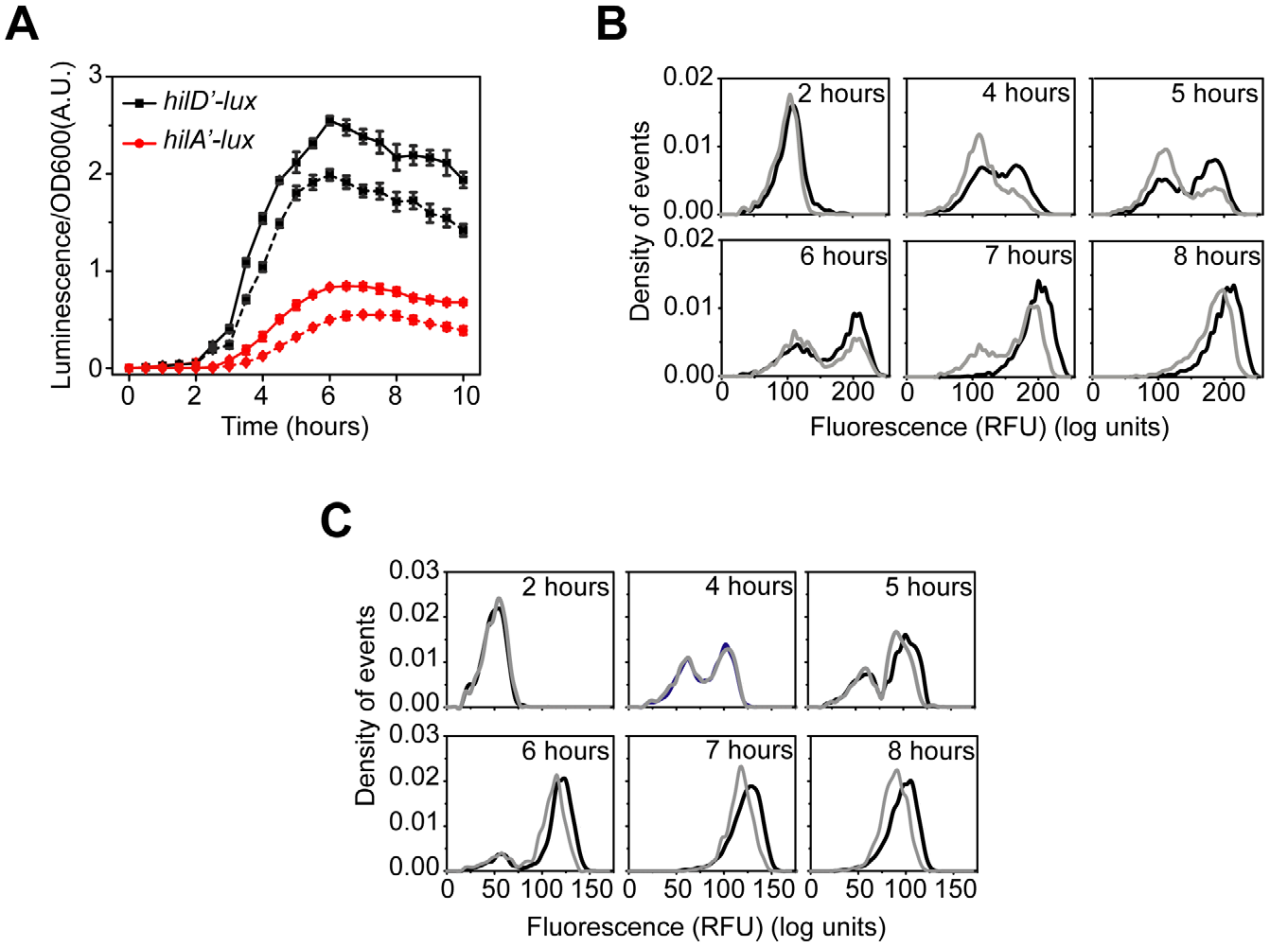
HilC and RtsA amplify SPI1 gene expression. (A) Comparison of time-course dynamics for P*_hilD_* (pSS074, black) and P*_hilA_* (pSS077, red) promoter activities in wild type (solid lines) and a Δ*hilC* Δ*rtsA* mutant (CR350, dashed lines) as determined using luciferase transcriptional reporters. (B and C) Comparison of P*_hilA_* (pSS055, B) and P*_hilD_* (pSS072, C) promoter activities in wild type (black) and a Δ*hilC* Δ*rtsA* mutant (CR350, grey) as determined using GFP transcriptional reporters and flow cytometry. Note that the loss of HilC and RtsA causes both a delay and decrease in P*_hilA_* promoter activity whereas it causes only a decrease in activity in the case of the P*_hilD_* promoter. Experiments were performed as described in **Figure 1**.

Unlike HilD, the HilC and RtsA proteins are not absolutely required for HilA expression. Yet, these two proteins can independently induce transcription from the P*_hilA_* promoter when constitutively expressed from an ectopic promoter [16]. To understand the role of these two proteins in regulating SPI1, we compared gene expression in wild type and a Δ*hilC* Δ*rtsA* mutant using the luciferase reporters (**Figure 3A**). Deleting these two regulators decreases the activity of the P*_hilD_* and P*_hilA_* promoters. Moreover, in the Δ*hilC* Δ*rtsA* mutant, there is also a delay in the induction of the P*_hilA_* promoter. This delay becomes more apparent when we normalize the luminescence measurements with respect to their maximal values (**Figure S3A**). When we measured gene expression at single-cell resolution using flow cytometry, we again observed a switch-like response in the Δ*hilC* Δ*rtsA* mutant (**Figure 3B**). The main difference relative to wild type was that the transition from the “off” to “on” state occurred more slowly in the absence of HilC and RtsA. Also, the activity of the P*_hilA_* promoter in the “on” state was lower in the Δ*hilC* Δ*rtsA* mutant than in wild type. With the P*_hilD_* promoter, we did not observe any change in the timing of promoter activation in the Δ*hilC* Δ*rtsA* mutant relative to wild type (**Figure 3C** **and S3A**). Rather, we observed only a decrease in the level of P*_hilD_* promoter activity associated with the “on” state. Similar results for both promoters are observed in the single deletion mutants, though the overall effect is small, indicating that HilC and RtsA additively contribute to SPI1 gene expression (**Figure S3B–E**). Based on these results, we conclude that HilC and RtsA serve two functions in the SPI1 circuit. First, HilC and RtsA amplify HilA and HilD expression, in the sense that HilA and HilD expression is reduced the absence of HilC and RtsA. Second, HilC and RtsA accelerate the transition of HilA expression from the “off” to the “on” state.

### HilE dampens SPI1 gene expression

We next investigated the role of HilE in the SPI1 gene circuit. HilE binds to HilD and prevents it from activating the P*_hilD_*, P*_hilC_*, P*_rtsA_*, and P*_hilA_* promoters [34]. As HilD is at the top of the SPI1 transcriptional cascade, HilE is able to repress the expression of all SPI1 genes. However, unlike the other regulators, HilE does not participate in a feedback loop, as its expression is not regulated by any SPI1 gene (data not shown). Rather, its expression is regulated by exogenous factors. For example, the type I fimbrial regulator, FimZ, increases HilE expression whereas the phosphoenolpyruvate phosphotransferase system (PTS) regulator, Mlc, represses it [23], [24], [35].

**Figure 4:**
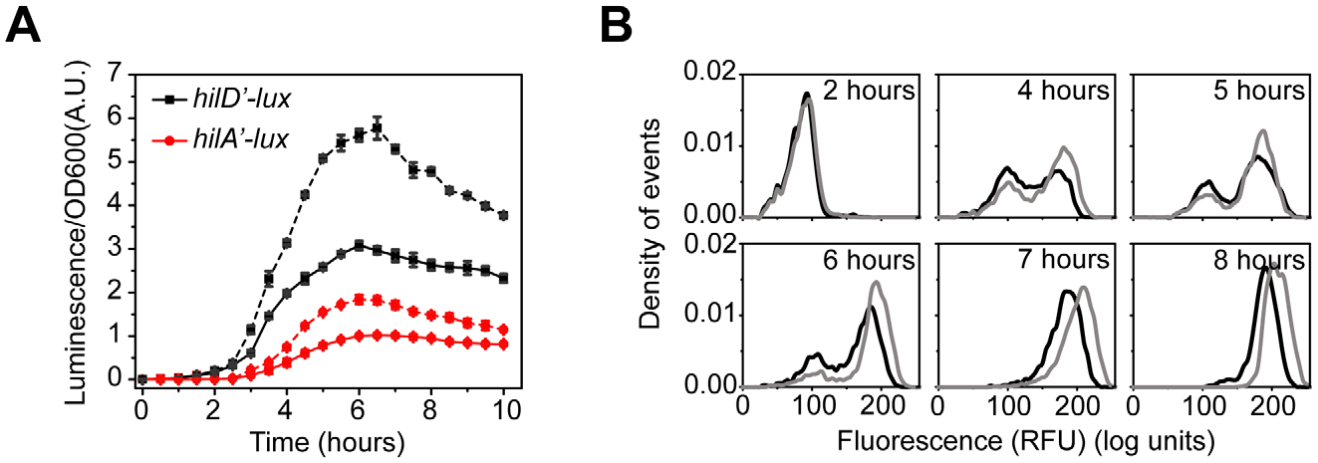
HilE dampens SPI1 gene expression. (A) Comparison of time-course dynamics for P*_hilD_* (pSS074, black) and P*_hilA_* (pSS077, red) promoter activities in wild type (solid lines) and a Δ*hilE* mutant (CR361, dashed lines) as determined using luciferase transcriptional reporters. (B) Comparison of P*_hilA_* (pSS055) promoter activities in wild type (black) and a Δ*hilE* mutant (CR361, grey) as determined using GFP transcriptional reporters and flow cytometry. Similar results are also observed with the P*_hilD_* promoter, though the phenotypic effect is much larger (**Figure S4**). Experiments were performed as described in **Figure 1**.

We compared gene expression using the luciferase assay in wild type and a Δ*hilE* mutant (**Figure 4A**). In the case of both the P*_hilD_* and P*_hilA_* promoters, we observed a roughly two-fold increase in promoter activity in the absence of HilE. However, we found that HilE did not affect the timing of activation for these two promoters (**Figure S4A**). Similar results were observed in the flow cytometry experiments for the P*_hilD_* and P*_hilA_* promoters (**Figure 4B** **and S4B**) and the P*_hilC_* and P*_rtsA_* promoters (data not shown). These data suggest that HilE serves to dampen SPI1 gene expression by reducing the maximal level of promoter activity.

### Computational analysis of SPI1 gene circuit

**Figure 5:**
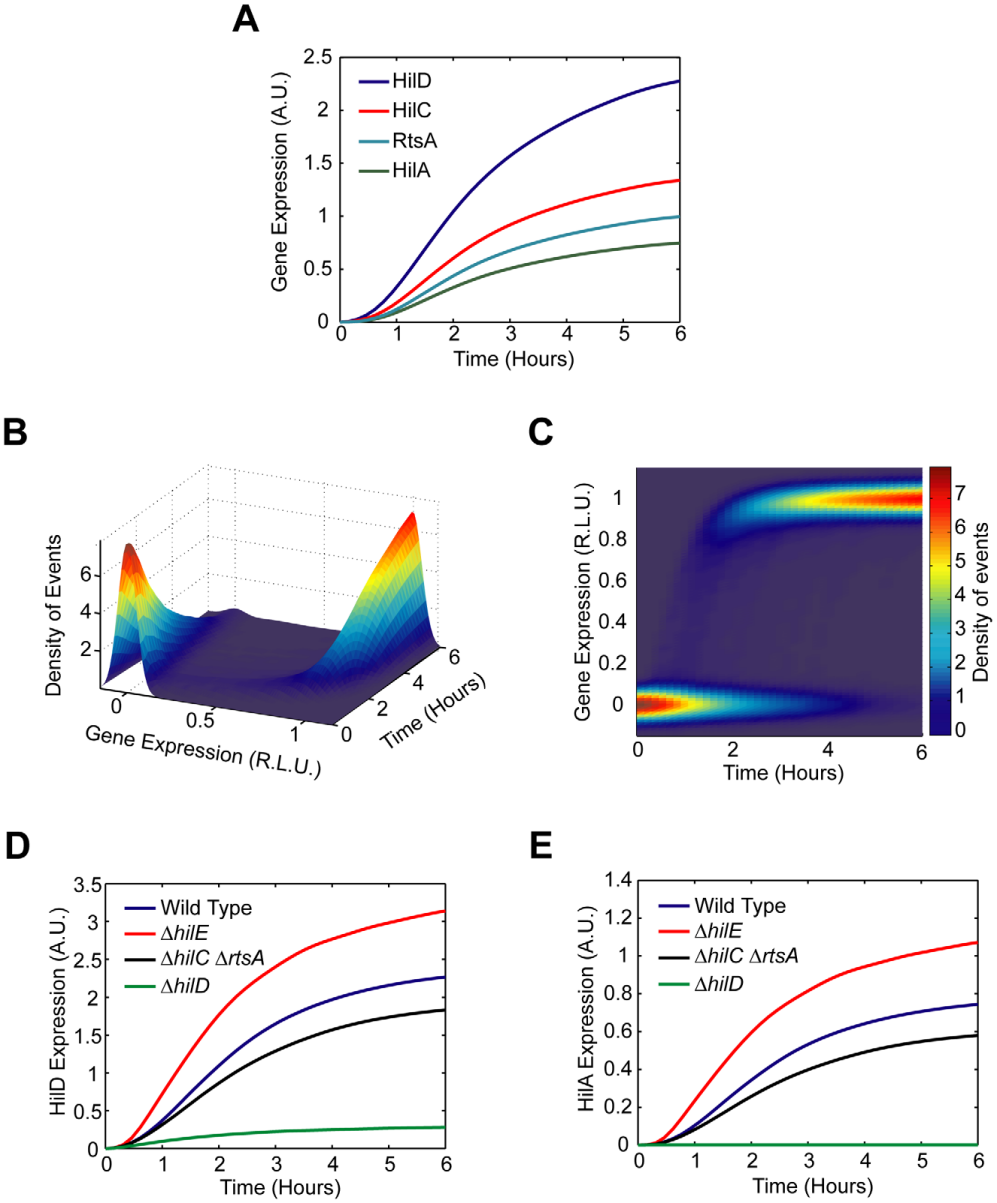
Mathematical model is able to accurately capture SPI1 gene expression dynamics both for wild type and key mutants. (A) Time-course simulation of HilD, HilC, RtsA, and HilA expression dynamics in wild-type cells. These results are the average of 1000 simulations. These simulations are meant to capture the population-level behavior of the circuit. (B) Time-course simulation of HilA expression at single-cell resolution. The expression values are normalized to one and plotted on a log scale. The expression values are given in relative log units (R.L.U.). Similar expression dynamics are also seen for HilD, HilC, and RtsA (see Matlab code provided as supplementary material). (C) Same results provided as a two-dimension heat plot, where the color intensity denotes the density of events. Note that the model captures the transient heterogeneity observed in our flow cytometry data where cells in both the “off” and “on” states are found at intermediate times. Panels A–C were generated from the same set of simulation runs. (D and E) Time-course simulation of HilD (D) and HilA (E) expression dynamics in wild type and Δ*hilD*, Δ*hilC* Δ*rtsA*, *and* Δ*hilE* mutants at population resolution. The results for each mutant were obtained from the average of 1000 simulations. Similar behavior is also seen at single-cell resolution. Mutants were simulated by setting the activity of the respective gene to zero in the model. A detailed description of the model is provided in the Materials and Methods.

The defining feature of the SPI1 gene circuit is the presence of three coupled positive feedback loops. An immediate question then is why are multiple loops present when most bacterial circuits employing feedback have just one. To explore this question in more detail, we constructed a simple mathematical model of the SPI1 gene circuit based on our understanding of how it functions (details provided in the Materials and Methods section). The model is qualitatively consistent with our experimental results, both with respect to the dynamics of HilD, HilC, RtsA, and HilA expression (**Figure 5A–C**) as well as the effects of mutations on HilD and HilA expression (**Figure 5D–E**) at both population and single-cell resolution.

In constructing this model, we assumed that asynchronous activation of the P*_hilD_* promoter in individual cells causes the transient heterogeneity observed in SPI1 gene expression. We specifically assumed that the P*_hilD_* promoter is activated at random times in individual cells, where the times are exponentially distributed. Otherwise, the model is entirely deterministic. To capture the heterogeneous response, we also needed to assume that the switch from the “off” to “on” state occurs rapidly in individual cells, more rapidly than what is observed in the population (**Figure 1B**). Otherwise, the cells will respond homogenously as differences in the timing of the activating signal in individual cells would be smoothed out due to the slow kinetics of the circuit. As our results demonstrate, this mechanism is sufficient for generating transient heterogeneity. In fact, if the P*_hilD_* promoter is activated in all cells at the same time or the kinetics of the switch are too slow, then the population behaves homogenously (**Figure S5A–B**). In the case of the P*_hilC_* promoter, we assumed that it was activated at the same time in all cells. While transient heterogeneity is observed in wild type cells (**Figure S1C**), the P*_hilC_* promoter behaves homogenously in a Δ*hilD* mutant (**Figure S2C**). Our model is also able to capture this behavior (**Figure S5C–D**).

Our goal in constructing this model was not simply to recapitulate our experimental results but rather to explore the behavior of the circuit by simulating it over a range of different parameter values. In particular, we employed the model to explore the roles of coupled positive feedback and HilE in regulating SPI1 gene expression. When performing this parametric analysis, we found it most informative to focus on the steady-state behavior of the SPI1 gene circuit. This enabled us to explore the effect of a limited number of model parameters two at a time and also bypass the issue of stochasticity. As a consequence, our analysis is confined to the parameters characterizing the regulatory topology of the circuit and not those defining the dynamics (e.g. degradation and protein-protein association/disassociation rates).

**Figure 6:**
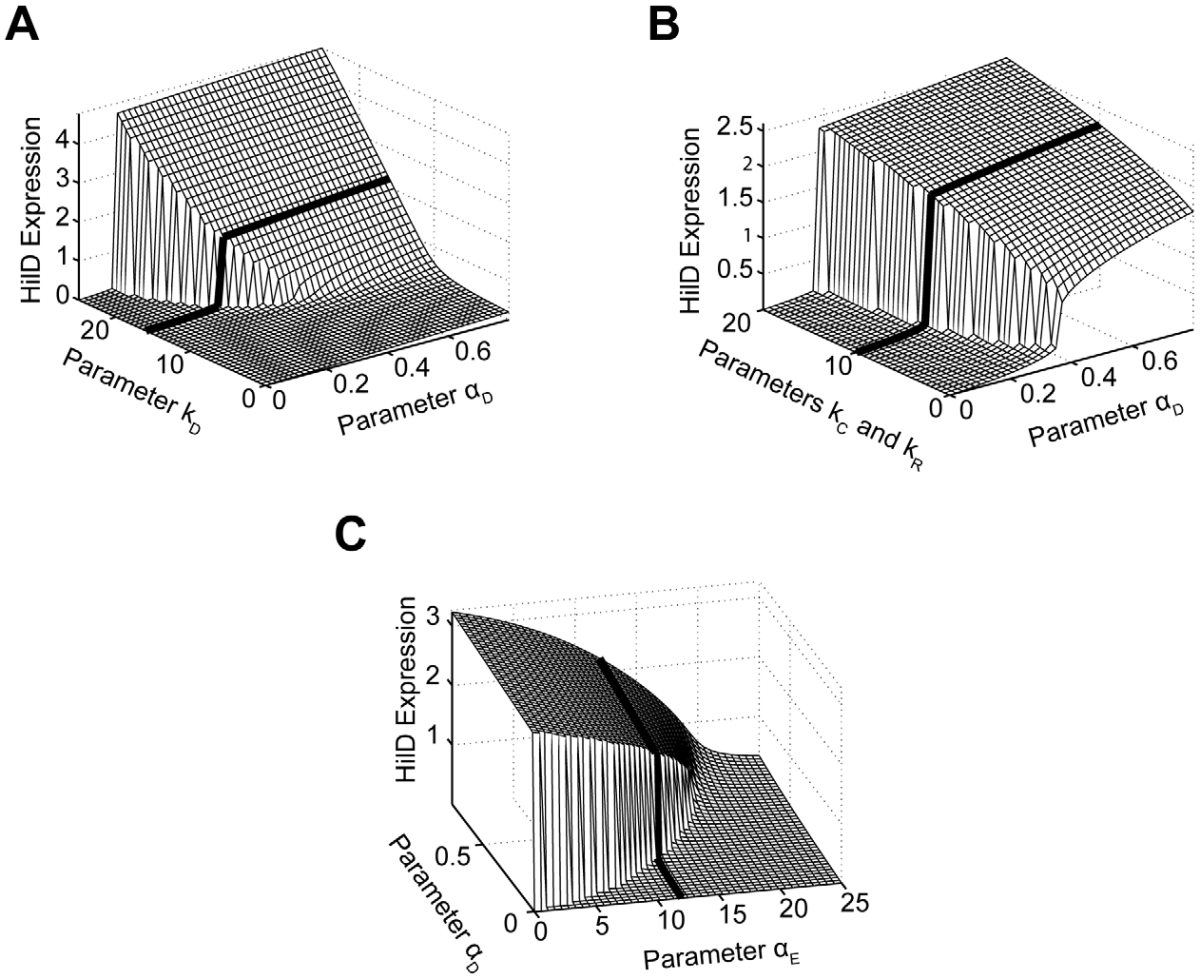
Parametric analysis of model predicts that SPI1 gene circuit functions as an amplifier and encodes an activation threshold. (A) Effect of positive feedback on HilD expression. Plot shows steady-state concentration of HilD as a function of the parameters 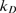 and 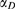. The parameter 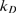 specifies the degree by which the SPI1 regulators - HilC, HilD, and RtsA - can activate HilD expression, effectively the strength of positive feedback on HilD expression. The parameter 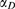 specifies the strength of the signal activating HilD expression. (B) Effect of HilC and RtsA on HilD expression. Plot shows the steady-state concentration of HilD as a function of the parameters 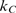, 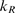, and 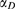. The parameters 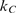 and 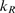 specify the degree by which the SPI1 regulators - HilC, HilD, and RtsA - can activate HilC and RtsA expression, respectively. In other words, these parameters set the strength of feedback on HilC and RtsA expression. In these simulations, the parameters 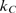 and 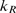 were both varied in tandem: the numerical values for the two are the same. (C) Effect of HilE on HilD expression. Plot shows the steady-state concentration of HilD as a function of the parameters 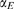 and 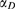. The parameter 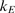 specifies the rate of HilE expression. Results for HilA are shown in **Figures S6A–C**. The black lines in the plots are used to denote the results obtained using the nominal parameters (aside from 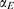). A detailed description of the model is provided in the Materials and Methods.

We first considered the role of positive feedback on HilD expression, given the central role of this SPI1 regulator. To perform this analysis, we varied the degree by which the SPI1 regulators - HilC, HilD, and RtsA - could activate HilD expression by simulating the model at different values for the parameter 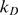. When interpreting these results, we found it informative to also vary the strength of the activating signal in our simulations, given by the parameter 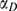 in the model. As shown in **Figure 6A**, HilD expression increases as the value of the parameter 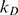 increases, equivalent to increasing the strength of the feedback on HilD expression. When this feedback is sufficiently strong, the response to the activating signal becomes discontinuous and switch-like. These results suggest that, in addition to amplifying the response, positive feedback may serve, along with HilE as described below, to endow the SPI1 circuit with an activation threshold. This threshold would ensure that SPI1 gene expression occurs only when a sufficiently strong activating signal is present. Moreover, the threshold decreases as the strength of the feedback increases, indicating that there is a tradeoff between the degree of amplification and the size of the threshold.

Next, we explored the effect of HilC and RtsA on SPI1 gene expression by varying the strength of their connectivity within the circuit. Specifically, we varied the degree by which the SPI1 regulators – HilD, HilC and RtsA - could enhance both HilC and RtsA gene expression, given respectively by the parameters 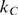 and 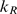 in the model. As HilC and RtsA both have a weaker effect on SPI1 gene expression than HilD, the degree of amplification is also less strong though the overall trend is the same (**Figure 6B**). Similar results are also obtained when the expression of only one protein is varied, though the effect then is even weaker (data not shown). These results suggest that HilC and RtsA serve to fine tune SPI1 gene expression. A useful analogy here is to consider the fine and coarse focusing knobs on a microscope, where HilC and RtsA provide the fine-tune control and HilD the coarse control. This may explain why HilC and RtsA have a significantly weaker effect on SPI1 gene expression than HilD as the circuit is more robust than one with three strong regulators in the sense that only a single regulator defines the behavior of the circuit whereas the others simply tune the output.

Last, we explored the effect of HilE on SPI1 gene expression. Unlike the other SPI1 regulators, HilE is not known to be involved in any feedback loops with the other SPI1 regulators. Rather, its expression is controlled by exogenous factors. In our simulations, we varied the rate of HilE expression, given by the parameter 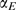 in the model. Consistent with its role as a negative regulator, HilE decreased both HilD and HilA expression in a dose-dependent manner (**Figures 6C** **and S6C**). In addition, when expressed at a sufficiently high rate, HilE effectively shuts off the expression of HilD and HilA, a result that we also observe experimentally (data not shown). Most notably, our model predicts that HilE sets the threshold for SPI1 activation - as the rate of HilE expression increases so does the threshold for activation and vice versa. The exogenous factors regulating HilE expression, therefore, may serve to tune this activation threshold. However, we note that HilE alone does not endow the SPI1 circuit with a threshold. Rather, the threshold results from the complex interplay between HilE and the HilD positive feedback loop (**Figure S6D**).

Taken together, these results allow us to assign putative function to the interacting regulators and associated feedback loops comprising the SPI1 gene circuit. When viewed as a whole, the circuit appears to serve two functions. The first is to place a threshold on SPI1 activation, ensuring that the assembly of the needle complex is initiated only in response to the appropriate combination of environmental and cellular cues. The second is to amplify SPI1 gene expression.

### Rewiring the SPI1 gene circuit

Our computational analysis predicts that the SPI1 gene circuit functions as a gene expression amplifier with a variable activation threshold. While our experimental results directly support the conclusion regarding gene amplification (**Figures 2** and **3**), the one concerning the activation threshold is not evident from our experimental results, and thus derives solely from analysis of the model. Therefore, to test this prediction regarding the threshold experimentally, we rewired the SPI1 gene circuit by replacing the P*_hilD_* promoter with the weaker P*_hilC_* promoter at its native chromosomal locus in an otherwise Δ*hilC* background. In this mutant, (ΔP*_hilD_*::P*_hilC_* Δ*hilC*), *hilD* is transcriptionally regulated in a manner similar to *hilC*. If the circuit does indeed function to place a threshold on activation, then we expect that this mutant will be unable to induce HilA expression if the activating signal for the P*_hilC_* promoter is too weak to overcome the threshold.

**Figure 7:**
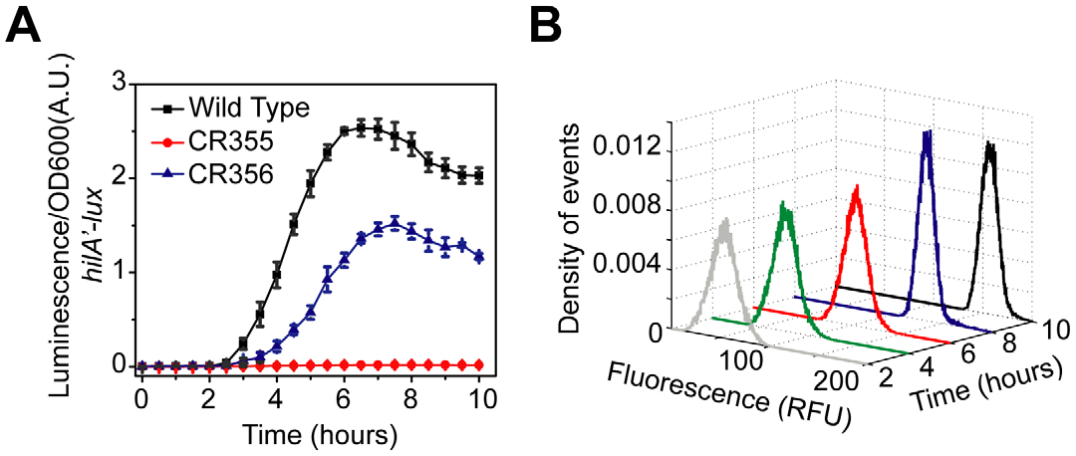
Rewiring SPI1 gene circuits demonstrates that HilE imposes threshold on activation. (A) Comparison of time-course dynamics for P*_hilA_* (pSS077) promoter activities in wild type (black), CR355 (ΔP*_hilD_*::P*_hilC_* Δ*hilC*, red) and CR356 (ΔP*_hilD_*::P*_hilC_* Δ*hilC* Δ*hilE*, blue) as determined using luciferase transcriptional reporters. In strain CR355, the P*_hilD_* promoter was replaced with the P*_hilC_* promoter in an otherwise Δ*hilC* background. In this strain, *hilD* is transcriptionally regulated in a manner identical to *hilC*. Strain CR356 is the same as CR355 except that it lacks HilE. (B) Dynamics of P*_hilA_* promoter activity in CR356 as determined using green fluorescent protein (GFP) transcriptional fusions and flow cytometry. Note that the activation of the P*_hilA_* promoter in CR356 is no longer switch-like but rather rheostatic in nature. Similar dynamics are seen with the P*_hilC_* promoter (**Figure S2C**). Experiments were performed as described in **Figure 1**.

We found the P*_hilA_* promoter is not active in this strain (**Figure 7A**), suggesting that the P*_hilC_* activating signal is too weak to overcome the threshold as hypothesized. If true, then according to our model, removing HilE should enable HilA expression as it sets the activation threshold. In agreement with our model predictions, we found that if the *hilE* gene is removed, then the P*_hilA_* promoter is active in a related strain (ΔP*_hilD_*::P*_hilC_* Δ*hilC* Δ*hilE*) (**Figure 7A**). In other words, by removing the threshold set by HilE, HilD is capable of inducing HilA expression when expressed from the weaker P*_hilC_* promoter. However, when the threshold is present, HilD is not expressed at sufficiently high levels to overcome this threshold.

When we measured gene expression in this strain (ΔP*_hilD_*::P*_hilC_* Δ*hilC* Δ*hilE*) using flow cytometry, we no longer observed the transient heterogeneity found in wild type. Rather, we found that the population responded homogeneously (**Figure 7B**). As we have previously noted, the input signal to the P*_hilC_* promoter is not switch-like but instead is homogenous and rheostatic in nature (**Figure S2C**). These results further support our hypothesis that the switch-like dynamics observed in wild type is due to asynchronous activation of the P*_hilD_* promoter (**Figure 2B**) and not intrinsic to the circuit. In particular, when *hilD* is expressed from the P*_hilC_* promoter then *hilA* expression is also not switch-like but instead homogenous and rheostatic. Thus, the characteristics of the output of the circuit match the input. In other words, the qualitative dynamics of the input driving *hilD* expression are also observed in the network output, namely *hilA* expression. Collectively, these results support our conclusion that the SPI1 gene circuit functions as a genetic amplifier with an activation threshold, where the circuit magnifies the activating signal only if this signal exceeds a defined threshold.

A remaining question concerns the uniqueness of the SPI1 regulators given their similarity to one another. Namely, to what degree are HilC, RtsA, and HilD interchangeable? Of the three, HilD is the most important as HilA is not expressed in its absence. In formulating the model, we needed to assume that HilD was dominant in the sense that it was required for activating HilA expression. We also needed to assume that HilD was necessary for establishing connectivity within the network, where it was again required for HilC- and RtsA-dependent activation of the P*_hilC_*, P*_hilD_*, and P*_rtsA_* promoters (see Materials and Methods for further details). HilC and RtsA, on the other hand, appear to play an ancillary role in regulating SPI1 gene expression. These two proteins simply tune gene expression in a HilD-dependent manner. One specific question then is whether this behavior is intrinsic to these proteins, as assumed in the model, or simply due to these proteins not being expressed at sufficiently high levels (as HilC and RtsA can independently activate SPI1 gene expression when over expressed).

To explore this issue in more detail, we rewired the SPI1 gene circuit by placing *hilC* under the control of the P*_hilD_* promoter. In this reciprocal design, we replaced the P*_hilC_* promoter with the P*_hilD_* promoter at its native chromosomal locus in an otherwise Δ*hilD* background (ΔP*_hilC_*::P*_hilD_* Δ*hilD*). The rationale behind this promoter replacement experiment was to see whether HilC alone could induce HilA expression if expressed from the P*_hilD_* promoter. As HilD is capable of inducing HilA expression in absence of HilC or RtsA, we reasoned that HilC may be able to do the same in the absence of HilD if it is transcribed in a manner similar to *hilD*. However, despite trying designs where different sections of the promoter region were replaced, we were unable to engineer a strain where the P*_hilA_* promoter was active in the absence of HilD (data not shown). These results lend credence to our hypothesis regarding HilD dominance used in formulating the model, namely that HilD is necessary for activating the SPI1 promoter under physiological conditions.

## Discussion

Using a combination of experimental and computational approaches, we found that the SPI1 gene circuit functions as a signal amplifier with an activation threshold. This virulence switch likely ensures that the SPI1 T3SS is assembled only when the bacterium has reached its target site for invasion, the distal small intestine [45]. *Salmonella* is thought to be able to determine its location within the host by sensing a number of environmental factors, key among them oxygen and osmolarity [28]. In addition to these environmental signals, SPI1 gene expression is also coordinated with other cellular processes such as motility and adhesion [19], [20], [21], [22], [23], [24]. The accumulated evidence to date, including the results from this study, indicates that HilD is the primary site for signal integration. According to our model, these activating signals, both intracellular cellular and environmental, initiate SPI1 gene expression by inducing the expression and activation of HilD through still unknown mechanisms. HilE, however, binds to HilD and inhibits its activity. Only when the activating signals are sufficiently strong is HilD expressed at a high enough level to overcome sequestration by HilE and activate the expression of the other SPI1 regulators – HilC, RtsA, and HilA - and also further induce its own expression. Once induced, HilC and RtsA serve to further amplify and also accelerate SPI1 gene expression. The result is a two-state switch with a defined activation threshold, defined in the sense that the threshold is set by the level of HilE expression and possibly other systems that function through HilD protein [44].

A notable feature of the SPI1 gene circuit is the presence of three, coupled positive feedback loops. At the most fundamental level, positive feedback amplifies the response to an external signal [46], [47]. It is also capable of effectively transforming a continuous input into a digital output when the feedback is sufficiently strong. In the context of bacterial gene circuits, positive feedback has most often been associated with multi-stable switches and cell population heterogeneity [43], [48]. What makes the SPI1 gene circuit particularly intriguing is that most bacterial systems utilizing positive feedback, at least those documented so far in the literature, possess only a single loop.

We first note that these additional feedback loops, namely the ones regulating the expression of HilC and RtsA, do not add redundancy to the circuit, as the loss of HilD effectively shuts off SPI1 gene expression. Rather, they serve to further amplify and accelerate SPI1 gene expression. *In vivo*, loss of either HilC or RtsA does not significantly attenuate intestinal invasion. Yet, loss of both does [16], indicating that the amplification or acceleration provided by these loops plays an important physiological role. Whether this role is simply to ensure that the SPI1 structural genes are expressed at sufficiently high levels or to provide a sharp activation threshold is still unknown.

Only a few studies to date, mostly focused on eukaryotic systems where this regulation is more common, have explored systems employing coupled positive feedback [49], [50], [51], [52]. In one notable theoretical study, the coupling of a slow and fast positive feedback loop was shown to yield a “dual-time” switch that is capable of being rapidly induced yet still is robust to fluctuations in the activating signal [49]. However, these properties are not obtained when two loops of the same type are coupled. While rapid induction is observed in SPI1 gene expression, there is no evidence to suggest that some loops are fast whereas others are slow. Furthermore, these loops do not operate synergistically in the sense that coupling in the SPI1 gene circuit does not engender new functions unattainable with just a single loop.

As the loops involving HilC and RtsA only additively contribute to the response, we imagine that the coupling in SPI1 may result instead from the piecewise evolution of the circuit. According to this model, HilC and RtsA were acquired to compensate for the inability of HilD alone to mediate a robust response. The motivation for this model comes from a recent study where a synthetic gene circuit coupling two weak positive feedback loops was engineered [53]. The authors found that their coupled circuit yielded a bistable response that, in the case of a single loop circuit, could be obtained only with an ultrasensitive activator even though the individual regulators in the coupled circuit lacked this behavior. Based on these results, the authors speculated that natural circuits could evolve using a similar approach - rather than evolve a circuit with a single regulator requiring precise biochemical properties, a more robust and facile solution may be obtained by simply linking together multiple regulators that alone lack the requisite properties. Similarly, others have shown that by changing the regulatory architecture of a circuit one can affect is behavior without commensurate changes in the underlying proteins [54], [55], [56]. We hypothesize that a similar process may have occurred with the SPI1 gene circuit. As such, this model provides one possible explanation as to why the circuit involves multiple feedback loops when one alone would suffice.

In a related study, we found that the gene circuit controlling the expression of type I fimbriae in *Salmonella* utilizes two coupled positive feedback loops [24]. In this system, the expression of the genes encoding the type I fimbriae is controlled by two regulators, FimY and FimZ. These two proteins form two coupled positive feedback loops and encode a logical AND gate or, alternatively, a coincidence circuit. A similar logic may also be also encoded within the SPI1 gene circuit. In particular, HilC is expressed in the absence of HilD. Moreover, the signals activating the P*_hilC_* promoter appear to be different than the ones activating the P*_hilD_* promoter, given their dissimilar dynamics. We are tempted therefore to speculate that, in addition to being an amplifier, the SPI1 gene circuit may also function as some sort of coincidence circuit, optimally expressing SPI1 genes only when the activating signals for both the P*_hilC_* and P*_hilD_* promoter are present. Coupled feedback in this case would reinforce the effect of these signals and further link the two. While such a model alone would not explain why multiple feedbacks loops are present in the SPI1 gene circuit, it may nonetheless provide one possible advantage for such a design.

In conclusion, we have been able to propose an integrated model for the regulation of SPI1 gene expression. While this system has been studied extensively, an integrated model of its regulation was previously lacking. Using a combination of experimental and computational analyses, we have been able to deconstruct this complex circuit and determine how the individual components contribute towards its integrated function. A key element in our analysis involved rewiring the SPI1 genetic circuit. As the kinetic parameters are unavailable and difficult to perturb, direct validation of our model remains an elusive challenge. However, by rewiring the circuit, we were nonetheless able to test a number of predictions from our mathematical model. Such an approach provides a powerful framework for integrating models with experimental data, particularly when parameters values are lacking or difficult to perturb. Finally, our results provide a detailed examination of a natural system employing coupled positive feedback, a mechanism of control that to date has primarily been investigated in eukaryotes.

## Materials and Methods

### Growth conditions

All experiments were performed in Luria-Bertani broth (LB) (10 g/L tryptone, 5 g/L yeast extract, and 10 g/L NaCl) unless otherwise specified. Bacterial strains were grown at 37°C except for strains carrying the temperature sensitive plasmids, pKD46 or pCP20, which were grown at 30°C as described previously [57]. Antibiotics were used at the following concentrations: ampicillin at 100 µg/mL; chloramphenicol at 34 µg/mL; kanamycin at 40 µg/mL, and tetracycline at 25 µg/mL.

### Bacterial strains and plasmids

All *Salmonella enterica* serovar Typhimurium strains used in this study are isogenic derivatives of strain ATCC 14028 (American Type Culture Collection) and are listed in **Table 1**. The strain CR349 (ΔSPI1::FRT Δ*rtsA5*) was made by first transducing the Δ(*invH-avrA*)*2916*::*cm* (called ΔSPI1::*cm*) allele from JS481 into the strain JS248 (Δ*rtsA5*) using P22HTint [58]. The chloramphenicol antibiotic resistance gene was then removed by introducing pCP20. To make the strain CR350 (Δ*hilC*::FRT Δ*rtsA5*), we first transduced the Δ*hilC*::*cm* allele from JS252 (Δ*hilC*::*cm*) into the strain JS248 (Δ*rtsA5*). The antibiotic resistance marker was then removed using pCR20. The strain CR351 (Δ*hilE*::*kan*) was made by replacing the *hilE* gene (genomic region 4763554–4764087) with the kanamycin resistance gene from pKD4 using λ-Red mediated homologous recombination [57].

**Table 1:** List of strains used in this study.

Strain	Genotype^a^	Source or Reference^b^
14028	Wild-type serovar Typhimurium	ATCC^c^
JS248	Δ*rtsA5*	[22]
JS252	Δ*hilC113::cm*	[22]
JS253	Δ*hilD114::cm*	[22]
JS481	Δ(*invH-avrA*)*2916*::*cm* (ΔSPI1::*cm*)	[16]
CR349	ΔSPI1::FRT Δ*rtsA5*	
CR350	Δ*hilC*::FRT Δ*rtsA5*	
CR351	Δ*hilE::kan*	
CR361	Δ*hilE*::FRT	
CR352	ΔP*_hilD_*::P*_hilC_*	
CR353	ΔP*_hilD_*::P*_hilC_* Δ*hilC::cm*	
CR354	ΔP*_hilD_*::P*_hilC_* Δ*hilC::cm* Δ*hilE::kan*	
CR355	ΔP*_hilD_*::P*_hilC_* Δ*hilC*::FRT	
CR356	ΔP*_hilD_*::P*_hilC_* Δ*hilC*::FRT Δ*hilE*::FRT	
CR357	ΔP*_hilC_*::P*_hilD_*	
CR358	ΔP*_hilC_*::P*_hilD_ hilD* RBS	
CR359	ΔP*_hilC_*::P*_hilD_* Δ*hilD::cm*	
CR360	ΔP*_hilC_*::P*_hilD_ hilD* RBS Δ*hilD::cm*	

a : All *Salmonella* strains are isogenic derivatives of serovar Typhimurium strain 14028.

b : Strains are from this study unless specified otherwise.

c : ATCC, American Type Culture Collection.

The strain CR352 (ΔP*_hilD_*::P*_hilC_*) was made using a two-step counter selection procedure involving the *tetRA* element from transposon Tn*10* [59]. In the first step, the P*_hilD_* promoter (genomic region 3017694–3017820) was replaced with the *tetRA* element using λ-Red mediated homologous recombination. The *tetRA* marker was then moved into a clean wild-type background (14028) by P22 transduction. Next, the *tetRA* element was replaced by the P*_hilC_* promoter (genomic region 3013780–3013010) using λ-Red mediated homologous recombination and a fusaric acid counter selection, as described previously [59]. The resulting strain, CR352 (ΔP*_hilD_*::P*_hilC_*), has the *hilD* gene with its native ribosome binding site under the control of P*_hilC_* promoter. The strain CR354 (ΔP*_hilD_*::P*_hilC_* Δ*hilC*::FRT Δ*hilE*::FRT) was made by P22 transduction, using the strains JS252 (Δ*hilC*::*cm*) and CR351 (Δ*hilE*::*kan*) as donors. The antibiotic resistance markers were removed by introducing pCP20. Similarly, we also constructed two strains, CR357 and CR358, where the P*_hilC_* promoter was replaced by the P*_hilD_* promoter. In the first design, CR357 (ΔP*_hilC_*::P*_hilD_*), the P*_hilC_* promoter (genomic region 3013780–3013010) was replaced with the P*_hilD_* promoter (genomic region 3017694–3017820) leaving the *hilC* ribosome binding site intact. In second design, CR358 (ΔP*_hilC_*::P*_hilD_ hilD* RBS), the upstream region of the *hilC* gene (genomic region 3013780–3013000) was replaced by the upstream region of the *hilD* gene (genomic region 3017694–3017830). All mutants were subsequently checked using primers that bound outside the region deleted. All chromosomal promoter replacements were verified by amplifying and sequencing the mutated regions.

All plasmids used in the study are listed in **Table 2**. Transcriptional fusions to the SPI1 promoters were made by cloning the promoter of interest upstream of either the green fluorescent protein (GFP) or the *luxCDABE* operon from *Photorhabdus luminescens* on a medium-copy plasmid [36], [37]. To construct the plasmid pSS098, pPROBE-*gfp* was digested with EcoRI and NheI and pPROTet.E was digested with EcoRI and AvrII. The *gfp* gene fragment from the digested pPROBE-gfp was then cloned into the digested pPROTet.E resulting in the plasmid pSS098. All constructs were sequenced prior to transformation in the wild-type and mutant strains.

**Table 2:** List of plasmids used in this study.

Plasmid	Characteristics	Region^a^	Reference^b^
pKD46	*bla* P*_BAD_ gam beto exo* pSC101 *oriTS*		[57]
pKD4	*bla* FRT *kan* FRT *oriR6K*		[57]
pCP20	*bla cat c*I857 λPRflp pSC101 *oriTS*		[68]
pPROBE-GFP	*kan gfp*[tagless] ori p15a		[38]
pPROBE-GFP[asv]	*kan gfp*[asv] ori p15a		[38]
pSS009	*kan luxCDABE* ori p15a		[37]
pSS052	*kan* P*_hilD_*-*gfp*[tagless] ori p15a	3017163–3017914	
pSS053	*kan* P*_hilC_*-*gfp*[tagless] ori p15a	3012997–3013773	
pSS054	*kan* P*_rtsA_*-*gfp*[tagless] ori p15a	4561763–4562111	
pSS055	*kan* P*_hilA_*-*gfp*[tagless] ori p15a	3018956–3019876	
pSS072	*kan* P*_hilD_*-*gfp*[asv] ori p15a	3017163–3017914	
pSS073	*kan* P*_hilC_*-*gfp*[asv] ori p15a	3012997–3013773	
pSS074	*kan* P*_hilD_*-*luxCDABE* ori p15a	3017163–3017914	
pSS075	*kan* P*_hilC_*-*luxCDABE* ori p15a	3012997–3013773	
pSS076	*kan* P*_rtsA_*-*luxCDABE* ori p15a	4561763–4562111	
pSS077	*kan* P*_hilA_*-*luxCDABE* ori p15a	3018956–3019876	
pPROTet.E	*cm* P_LTetO-1_ ori ColE1		Stratagene
pSS098	*cm* P_LTetO-1_-*gfp*[tagless] ori ColE1		

a : Genomic region used to construct transcriptional fusion based on *Salmonella enterica* serovar Typhimurium LT2 genome sequence [69].

b : Plasmids are from this study unless specified otherwise.

### Fluorescence measurements

Cultures were first grown overnight in LB medium lacking salt under vigorous shaking at 37°C (SPI1 repressing conditions) and then sub-cultured 1∶1000 into fresh LB medium (with salt) and grown statically in test tubes at 37°C for 12 hours [34], [60]. A 100 µL aliquot of each culture was then transferred to a 96-well microplate, and fluorescence and absorbance (OD600) were measured using a Tecan Safire2 microplate reader. The fluorescence readings, given in terms of relative fluorescence units (RFU), were normalized to the OD600 absorbance to account for cell density.

For single-cell fluorescence measurements, overnight cultures were first grown under SPI1-repressing conditions at 37°C. The cells were then sub-cultured to an OD of 0.05 into fresh LB medium (with salt) and grown statically at 37°C. Samples were collected at different time points by resuspending them in phosphate buffered saline (PBS) with 34 µg/mL chloramphenicol in order to arrest translation and then storing on ice. All fluorescent-activated cell sorting (FACS) experiments were performed on a BD LRS II system from BD Biosciences. Data extraction and analysis for the FACS experiments was done using FCS Express Version 3 (De Novo Software). For all FACS experiments, fluorescence values of 30,000 events were recorded and reported as a histogram.

In the flow cytometry experiments involving the P*_hilC_* and P*_hilD_* promoters, we used destabilized GFP transcriptional fusions where the sequence AANDENYAASV was appended to the C-terminus of the protein. This tag reduces the half life of GFP from approximately 24 hours to 110 minutes [38], [61]. The reason that we needed to employ destabilized GFP is that P*_hilC_* and P*_hilD_* promoters are partially active even when the cells are grown in SPI1-repressing conditions. As a consequence, we were unable to observe the “off” to the “on” transition using “tagless” GFP. We did not run into similar problems with the P*_hilA_* and P*_rtsA_* promoters and consequently used transcriptional fusions to “tagless” GFP. Qualitatively similar results are obtained when using destabilized GFP transcriptional fusions to these promoters (data not shown).

### Luminescence measurements

For time-course luminescence experiments, cultures were grown overnight at 37°C in SPI1-repressing conditions. The overnight cultures were then sub-cultured to an OD of 0.05 into fresh LB medium (with salt). A 100 µL aliquot of each culture was then transferred to a 96-well microplate. This is denoted by time 0 h in our kinetic luminescence experiments. In addition, 20 µL of mineral oil was also added to the well to prevent evaporation [62]. The cells were then grown statically at 37°C, and luminescence and absorbance (OD600) readings were taken every 5 minutes using a Tecan Safire2 microplate reader. The luminescence readings, given in terms of relative light units (RLU), were normalized to the OD600 absorbance to account for cell density. Three independent experiments were performed on separate days. For each experiment, six samples were tested. The average values and standard deviations are reported.

### Model description

The major assumptions used in formulating the model are enumerated below.

1. In formulating the model, we focused solely on the interacting SPI1 regulators - HilC, HilD, HilE, and RtsA - and their role in regulating *hilA* expression. In particular, we ignored the effects of additional external regulators [17], [44]. These external factors were accounted for implicitly in the model through our choice of the kinetic parameters. In other words, we assumed that there are no additional feedback loops beyond those detailed in **Figure 1A**. As a consequence, we treated these external regulators as constant inputs into the model. The validity of this hypothesis is debatable, though there is insufficient evidence at this time to consider any reasonable alternatives. We also did not include the downstream SPI1 regulators – InvF and SicA – in the model. These downstream regulators do not appear to affect HilA expression. Rather, they are thought to regulate the timing of expression of the proteins comprising the SPI1 needle complex and the secreted effectors [29], [41], [63]. In these regards, the model focuses only on initiation and ignores assembly and secretion. It also does not account for the decrease in SPI1 gene expression when cells enter stationary phase (**Figure 1B**).
2. The model does not account for negative regulation by HilA and SprB. HilA, in particular, negatively regulates its own expression by apparently binding to the P*_hilA_* promoter and repressing transcription [64]. Likewise, SprB, a transcription factor from the LuxR/UhaP family that is positively regulated by HilA, appears to bind to the HilD promoter and weakly repress its activity [65]. Inclusion of these negative feedback loops does not substantively affect the results from our model and, for simplicity, we chose to ignore them in the model.
3. The model does not distinguish between transcription and translation. Both are lumped together in a single step. As a consequence, the rate of protein synthesis is assumed to be linearly proportional to the concentration of mRNA within the cell. Our justification for this assumption is that, based on a number of unpublished observations, we believe that the regulation of HilD occurs primarily either at the transcriptional or the post-translational level (i.e. the level of HilD protein).
4. HilC, HilD, and RtsA are all AraC-like transcription factors and likely function only in the dimeric form. In the model, we assume for simplicity that the dimers form spontaneously and are stable (i.e. the dimerization reaction is irreversible). As a consequence, the model does not distinguish between the monomeric and dimeric forms; all protein is assumed to be in the dimeric form. We also do not account for the possible formation of heterodimers.
5. HilC and RtsA can independently induce HilA expression [16]. Yet, in the absence of HilD, HilA is not expressed even though *hilC* is transcribed (albeit at reduced levels). To account for HilD dominance (or rather dominant epistasis) in the model, we needed to assume that the SPI1 promoters have two binding sites with occupancy of both required for transcription. We specifically assumed that one site is highly specific for HilD with only weak affinity for HilC and RtsA. This first binding site establishes dominance as it effectively probes for whether HilD is present in the cell. Moreover, because of its high affinity, HilD will occupy this site even when expressed at low levels. Due to their weak affinity, neither HilC nor RtsA will occupy this site under physiological conditions. However, when over expressed, the elevated concentrations of these proteins will compensate for their weak affinity for this site, allowing them to bind. The second site, on the other hand, has moderate affinity for all three regulators (with the affinity for HilD still the highest) and serves to tune expression in proportion to their aggregate concentration. Other alternatives are possible, though this model for promoter regulation offers perhaps the simplest mechanism to explain HilD dominance consistent with what we already know about SPI1 gene expression. Moreover, others have found that the SPI1 promoters contain multiple binding sites for the HilC, HilD, and RtsA [31], [32], so this assumption is not entirely implausible. Lastly, we note that while HilD dominance has been documented previously only in the case of the P*_hilA_* promoter, our data suggests that it also extends to the P*_hilC_*, P*_hilD_*, and P*_rtsA_* promoters as detailed below.
6. The most speculative aspect of the model concerns the mechanism for activation of the SPI1 promoters – P*_hilA_*, P*_hilC_*, P*_hilD_*, and P*_rtsA_* - by HilC, HilD, and RtsA. In the model, we assume that all four promoters have the same two binding sites, one highly specific for HilD and the other much less so (see Assumption 5). While there is no mechanistic data to support this hypothesis, we have found that the promoter activities are linearly proportional to one another when we compared them at varying levels of NaCl induction and in different genetic backgrounds (**Figure S7**). The simplest explanation for this linear correlation is that all four promoters have the same two binding sites. As a consequence, we used the same mathematical expressions and parameters to model occupancy of the P*_hilA_*, P*_hilC_*, P*_hilD_*, and P*_rtsA_* promoters by the SPI1 regulators. Aside from our supporting data, we significantly reduce the number of free parameters in the model by invoking this assumption.
7. The model assumes that HilE not only binds and inhibits HilD but also promotes its degradation. While there is no experimental data to support such a mechanism, we found it necessary to match our experimental results for the Δ*hilE* mutant. In the absence of such a mechanism, we found that the steady-state concentrations of HilD and HilA were not affected by HilE, a result contrary to experimental observations.
8. The model assumes that the transient heterogeneity observed in the gene expression data is due solely to asynchrony in the timing of the activation signal. To model this behavior, we assumed that the P*_hilD_* promoter is activated at random times in individual cells, where the activation times are exponentially distributed. In the case of the P*_hilC_* promoter, we assumed that it is activated in a deterministic manner. For simplicity, we assumed that both promoters have, on average, the same activation kinetics. Beyond asynchrony in the timing of activation, we do not believe that noise arising from any number of possible sources plays a critical role in SPI1 gene expression beyond introducing variability in the gene expression measurements (see below).
9. To qualitatively compare the simulation results with our flow cytometry data, we employed density estimation using a Gaussian kernel with fixed bandwidth. This method replaces each data point with a Gaussian basis function of constant variance. While this method is typically used to smooth data, namely to approximate a discrete histogram with a continuous function, we employed it to artificially introduce noise into our model. Our motivation was simply to obtain a better qualitative fit to the flow cytometry data where, aside from the heterogeneity, we observed variable gene expression in individual cells. While we do not believe this variability is significant for understanding how the circuit functions, we nonetheless attempted to capture it in our model. As we do not know the origins of this variability (e.g. stochastic gene expression, measurement error, etc), we simply assumed that there was an additive Gaussian noise term in the model, effectively what density estimation does.

We note that Mande and coworkers previously published a mathematical model of the SPI1 gene circuit [66], [67]. While there is substantial overlap between their model and ours, the Mande model does not account for the critical role of positive feedback on HilD expression, a key finding in our experimental investigations. More significantly, their model does not include HilE. As a consequence, the major conclusion drawn from the analysis of our model regarding the activation threshold cannot be obtained from theirs.

### Model equations

The governing equations for the model are the following:
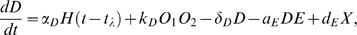
(1)

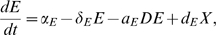
(2)

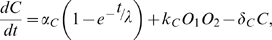
(3)

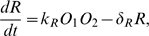
(4)

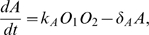
(5)

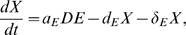
(6)

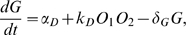
(7)where 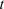 denotes time and the state variable 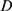 denotes the concentration of HilD, 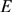 the concentration of HilE, 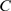 the concentration of HilC, 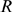 the concentration of RtsA, 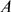 the concentration of HilA, 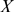 the concentration of the HilE-HilD complex, and 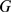 the concentration of the luciferase reporter for the P*_hilD_* promoter. We included this last state variable, 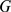, to better match the model to our experimental data. Otherwise, we needed to account for the fraction of HilD bound to HilE (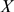) and the associated differences in the stabilities of the respective moieties. The variable 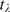 is used to denote an exponentially distributed random variable with a rate parameter 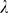 and the function 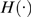 is used to denote the Heaviside step function. The occupancy state of the two respective binding sites within the SPI1 promoters are given by the following equilibrium expressions
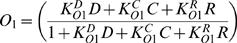
(8)and
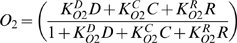
(9)The parameter definitions and nominal values are given in **Table 3**.

**Table 3:** Parameter definitions and nominal values.

Parameter	Description	Value^a^
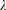	Initiation rate for P*_hilD_* and P*_hilC_* promoter	2.0 hr^−1^
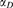	Basal activity for P*_hilD_* promoter	1.2 N hr^−1^
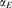	Basal activity for P*_hilE_* promoter	12.0 N hr^−1^
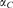	Basal activity for P*_hilC_* promoter	0.4 N hr^−1^
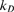	Activity for P*_hilD_* promoter	16.0 N hr^−1^
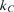	Activity for P*_hilC_* promoter	10.0 N hr^−1^
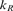	Activity for P*_rtsA_* promoter	8.0 N hr^−1^
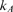	Activity for P*_hilA_* promoter	6.0 N hr^−1^
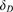	HilD degradation/dilution rate	4.0 hr^−1^
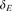	HilE degradation/dilution rate	8.0 hr^−1^
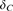	HilC degradation/dilution rate	4.0 hr^−1^
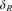	RtsA degradation/dilution rate	4.0 hr^−1^
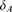	HilA degradation/dilution rate	4.0 hr^−1^
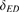	HilE-HilD degradation/dilution rate	16.0 hr^−1^
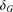	Reporter degradation/dilution rate	4.0 hr^−1^
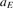	Association rate of HilD and HilE	8.0 N^−1^ hr^−1^
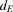	Disassociation rate of HilE-HilD complex	8.0 hr^−1^
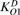	Equilibrium constant for HilD- 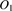 complex	10.0 N^−1^
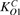	Equilibrium constant for HilC- 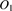 complex	0.001 N^−1^
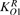	Equilibrium constant for RtsA- 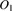 complex	0.001 N^−1^
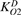	Equilibrium constant for HilD- 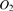 complex	1.0 N^−1^
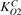	Equilibrium constant for HilC- 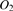 complex	0.1 N^−1^
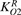	Equilibrium constant for RtsA- 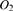 complex	0.1 N^−1^
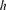	Bandwith for density estimation	0.05 N

a We are unable to assign absolute concentrations units to the parameter values. As a consequence, we report the parameters in terms of dimensionless concentration units, denoted by N.

In our simulations, we first generated the pseudo random variable 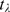 and then simulated the model using this value. The value for 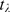 denotes the time when the P*_hilD_* promoter is activated in an individual cell. To model this transition, we employed the Heaviside step function, which has a value zero when the argument is negative and one when positive. Thus, when induced, the P*_hilD_* promoter undergoes a step-like increase in activity. We then repeated this procedure multiple times in order to gather statistics for an ensemble of cells.

With regards to the model parameters, insufficient data are available to accurately and uniquely estimate them. However, as our goal was simply to construct a model that captured the general trends observed in the data, we simply choose numerical values for the parameters that provided a good qualitative fit. In these regards, the model is only semi-quantitative given the subjective basis of our parameterization. That said, the model captures our current understanding of the SPI1 gene circuit and provides a reasonable fit to the data as documented in the main text.

### Numerical solution

The set of coupled ordinary differential equations comprising the model were solved in Matlab 7.2 (The Mathworks, http://www.mathworks.com) using the *ode15s* routine where the initial conditions of all state variables where set to zero. To account for random initiation times, the model was simulated 1000 times using the built-in random number generator. The Matlab m-file used to generate the figures is provided as supplemental information.

## Supporting Information

Figure S1 SPI1 gene expression dynamics in wild type. (A) Normalized activities of the P*_hilD_*, P*_hilC_*, P*_rtsA_*, and P*_hilA_* promoters in wild-type cells. The data from Figure 1B were normalized with respect to their maximal value. (B–D) Dynamics of P*_hilD_* (pSS072, B), P*_hilC_* (pSS073, C), and P*_rtsA_* (pSS054, D) promoter activities in wild-type cells as determined using green fluorescent protein (GFP) transcriptional fusions and flow cytometry. (E) Dynamics of the constitutively active PL*_tetO-1_* (pSS098) promoter in wild type cells as determined using GFP and flow cytometry. Note that the dynamics of this promoter are continuous and not switch like. Experiments were performed as described in Figure 1.
(0.35 MB PDF)

Figure S2 Activation of the SPI1 gene circuit. (A). Comparison of time-course dynamics of P*_hilD_* (pSS072) promoter activities in wild type (black) and a ΔSPI1 Δ*rtsA* mutant (CR349, grey) as determined using green fluorescent protein (GFP) transcriptional fusions and flow cytometry. (B) Comparison of P*_hilC_* (pSS075, black) and P*_rtsA_* (pSS076, red) promoter activities in wild type (solid lines) and a Δ*hilD* mutant (JS253, dashed lines). Note that the P*_rtsA_* promoter is off in the absence of HilD. (C) Dynamics of P*_hilC_* (pSS073) promoter activities in a Δ*hilD* mutant (JS253) as determined using green fluorescent protein (GFP) transcriptional fusions and flow cytometry. Note that, in the absence of HilD, the activation of the P*_hilC_* promoter is no longer switch-like but instead continuous. Experiments were performed as described in Figure 1.
(0.23 MB PDF)

Figure S3 HilC and RtsA amplify SPI1 gene expression in an additive manner. (A) Normalized P*_hilD_* and *_PhilA_* promoter activity in wild type (solid) and Δ*hilC* Δ*rtsA* (dashed) mutant. The data from Figure 3A were normalized with respect to their maximal value. (B) Comparison of time-course dynamics of P*_hilA_* (pSS077) promoter activities in wild type and Δ*rtsA* (JS248), Δ*hilC* (JS252), Δ*hilC* Δ*rtsA* (CR350), and Δ*hilD* (CR253) mutants as determined using luciferase transcriptional reporters. (C) Comparison of P*_hilA_* (pSS055) promoter activities in wild type (black) and Δ*hilC* (JS252, red) and Δ*rtsA* (JS248, grey) mutants as determined using green fluorescent protein (GFP) transcriptional fusions and flow cytometry. (D) Comparison of time-course dynamics of P*_hilD_* (pSS074) promoter activities in wild type and Δ*rtsA* (JS248), Δ*hilC* (JS252), Δ*hilC* Δ*rtsA* (CR350), and Δ*hilD* (CR253) mutants as determined using luciferase transcriptional reporters. (E) Comparison of P*_hilD_* (pSS072) promoter activities in wild type (black) and Δ*hilC* (JS252, red) and Δ*rtsA* (JS248, grey) mutants as determined using green fluorescent protein (GFP) transcriptional fusions and flow cytometry. Experiments were performed as described in Figure 1.
(0.39 MB PDF)

Figure S4 HilE negatively regulates HilD expression. (A) Normalized P*_hilD_* and P*_hilA_* promoter activities in wild type (solid) and Δ*hilE* (dashed) mutant. The data from Figure 4A was normalized to one for each strain. (B) Comparison of P*_hilD_* (pSS072) promoter activities in wild type (black) and Δ*hilE* (CR361, gray) mutant as determined using green fluorescent protein (GFP) transcriptional fusions and flow cytometry. Experiments were performed as described in Figure 1.
(0.12 MB PDF)

Figure S5Factors determining SPI1 switch. (A) HilA expression at single-cell resolution when activation of the P*_hilD_* promoter is deterministic. In these simulations, we changed the equation for HilD in the model to:
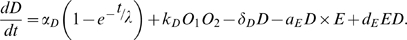
All other equations in the model are unchanged. (B) HilA expression at single-cell resolution when the kinetic parameters are reduced by a factor of ten. In our simulations, we accomplished this by reducing λ by a factor of ten and rescaling time by a factor of ten. (C–D) Comparison of HilC expression at single-cell resolution in wild type (C) and Δ*hilD* mutant (D). Figures are given as two-dimension heat plots, where the color intensity denotes the density of events. The results for each plot were obtained from 1000 simulations. The expression values are normalized to one and plotted on a log scale. The expression values are given in relative log units (R.L.U.). Mutants were simulated by setting the activity of the respective gene to zero in the model. A detailed description of the model is provided in the Materials and Methods.(0.18 MB PDF)

Figure S6 Parametric analysis of model predicts that SPI1 gene circuit functions as an amplifier and encodes a hard activation threshold. (A) Effect of HilD positive feedback on HilA expression. Plot shows steady-state concentration of HilD as a function of the parameters *k_D_* and *α_D_*. The parameter *k_D_* specifies the degree by which the SPI1 regulators - HilC, HilD, and RtsA - can activate HilD expression, effectively the strength of positive feedback on HilD expression. The parameter *α_D_* specifies the strength of the signal activating HilD expression. (B) Effect of HilC and RtsA on HilA expression. Plot shows the steady-state concentration of HilD as a function of the parameters *k_C_*, *k_R_*, and *α_D_*. The parameters *k_C_* and *k_R_* specify the degree by which the SPI1 regulators - HilC, HilD, and RtsA - can activate HilC and RtsA expression, respectively. In other words, these parameters set the strength of feedback on HilC and RtsA expression. In these simulations, the parameters *k_C_* and *k_R_* were both varied in tandem: the numerical values for the two are the same. (C) Effect of HilE on HilA expression. Plot shows the steady-state concentration of HilD as a function of the parameters *α_E_* and *α_D_*. (D–E) Effect of HilE and HilD positive feedback on HilD (D) and HilA (E) expression. Plots shows the steady-state concentrations of HilD and HilA as a function of the parameters *α_E_* and *k_D_*. The black lines in the plots (A–C) are used to denote the results obtained using the nominal parameters (aside from *α_E_*). A detailed description of the model is provided in the Materials and Methods.
(0.79 MB PDF)

Figure S7 SPI1 promoter activities are linear correlated to one another at varying levels of induction and in different mutants. (A) Correlation of P*_hilD_* (pSS052) promoter activity with P*_hilA_* (pSS055), P*_hilC_* (pSS053), and P*_rtsA_* (pSS054) promoter activities. To induce SPI1 gene expression, cells were first grown overnight in LB/no salt and then sub-cultured into fresh LB at varying concentrations of NaCl to an OD of 0.05 and grown statically for 15 hours. Individual experiments used to construct correlations are given in Panels B–E. (B–E) Comparison of P*_hilD_* (pSS052, B), P*_hilC_* (pSS053, C), P*_hilA_* (pSS055, D), and P*_rtsA_* (pSS054, E) promoter activities at varying concentration of NaCl and in different mutant backgrounds as determined using GFP transcriptional reporters. Fluorescence values were normalized with the OD600 absorbance to account for cell density. Data is the average of three independent experiments. Error-bars denote standard deviation.
(0.36 MB PDF)
