# Correction: The Role of Coupled Positive Feedback in the Expression of the SPI1 Type Three Secretion System in *Salmonella*

**DOI:** 10.1371/annotation/df7e26bc-4c62-43b4-865f-a39274d98ab3

**Published:** 2010-08-05

**Authors:** Supreet Saini, Jeremy R. Ellermeier, James M. Slauch, Christopher V. Rao

A file was erroneously omitted from the list of Supporting Information. Its description is "Matlab file used to generate all figures in manuscript" and can be viewed here: 
