# Supplementary material for: The Role of Coupled Positive Feedback in the Expression of the SPI1 Type Three Secretion System in Salmonella
Source: PLoS Pathog. 2010 Jul 29;6(7):e1001025. doi: 10.1371/journal.ppat.1001025 (PMC2912647; doi:10.1371/journal.ppat.1001025)
Supplement: Figure S1 — SPI1 gene expression dynamics in wild type. (A) Normalized activities of the PhilD, PhilC, PrtsA, and PhilA promoters in wild-type cells. The data from Figure 1B were normalized with respect to their maximal value. (B–D) Dynamics of PhilD (pSS072, B), PhilC (pSS073, C), and PrtsA (pSS054, D) promoter activities in wild-type cells as determined using green fluorescent protein (GFP) transcriptional fusions and flow cytometry. (E) Dynamics of the constitutively active PLtetO-1 (pSS098) promoter in wild type cells as determined using GFP and flow cytometry. Note that the dynamics of this promoter are continuous and not switch like. Experiments were performed as described in Figure 1. (0.35 MB PDF) [file ppat.1001025.s001.pdf]

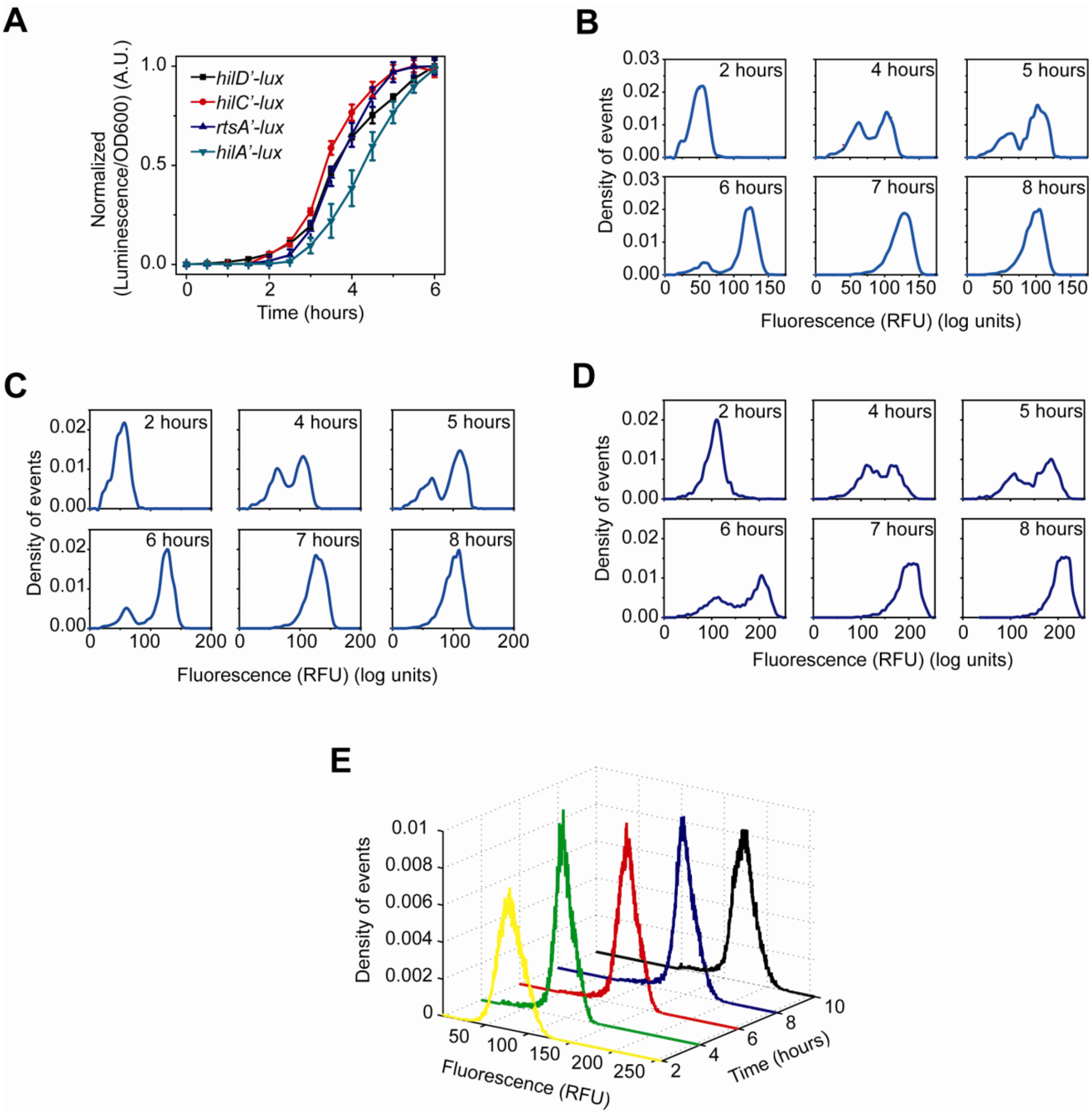

Figure S1. SPI1 gene expression dynamics in wild type.

(A) Normalized activities of the P_{hilD}, P_{hilC}, P_{rtsA}, and P_{hilA} promoters in wild-type cells. The data from Figure 1B were normalized with respect to their maximal value. (B-D) Dynamics of P_{hilD} (pSS072, B), P_{hilC} (pSS073, C), and P_{rtsA} (pSS054, D) promoter activities in wild-type cells as determined using green fluorescent protein (GFP) transcriptional fusions and flow cytometry. (E) Dynamics of the constitutively active $P_{LtetO\text{-}1}$ (pSS098) promoter in wild type cells as determined using GFP and flow cytometry. Note that the dynamics of this promoter are continuous and not switch like. Experiments were performed as described in Figure 1.
