# Supplementary material for: The Role of Coupled Positive Feedback in the Expression of the SPI1 Type Three Secretion System in Salmonella
Source: PLoS Pathog. 2010 Jul 29;6(7):e1001025. doi: 10.1371/journal.ppat.1001025 (PMC2912647; doi:10.1371/journal.ppat.1001025)
Supplement: Figure S2 — Activation of the SPI1 gene circuit. (A). Comparison of time-course dynamics of PhilD (pSS072) promoter activities in wild type (black) and a ΔSPI1 ΔrtsA mutant (CR349, grey) as determined using green fluorescent protein (GFP) transcriptional fusions and flow cytometry. (B) Comparison of PhilC (pSS075, black) and PrtsA (pSS076, red) promoter activities in wild type (solid lines) and a ΔhilD mutant (JS253, dashed lines). Note that the PrtsA promoter is off in the absence of HilD. (C) Dynamics of PhilC (pSS073) promoter activities in a ΔhilD mutant (JS253) as determined using green fluorescent protein (GFP) transcriptional fusions and flow cytometry. Note that, in the absence of HilD, the activation of the PhilC promoter is no longer switch-like but instead continuous. Experiments were performed as described in Figure 1. (0.23 MB PDF) [file ppat.1001025.s002.pdf]

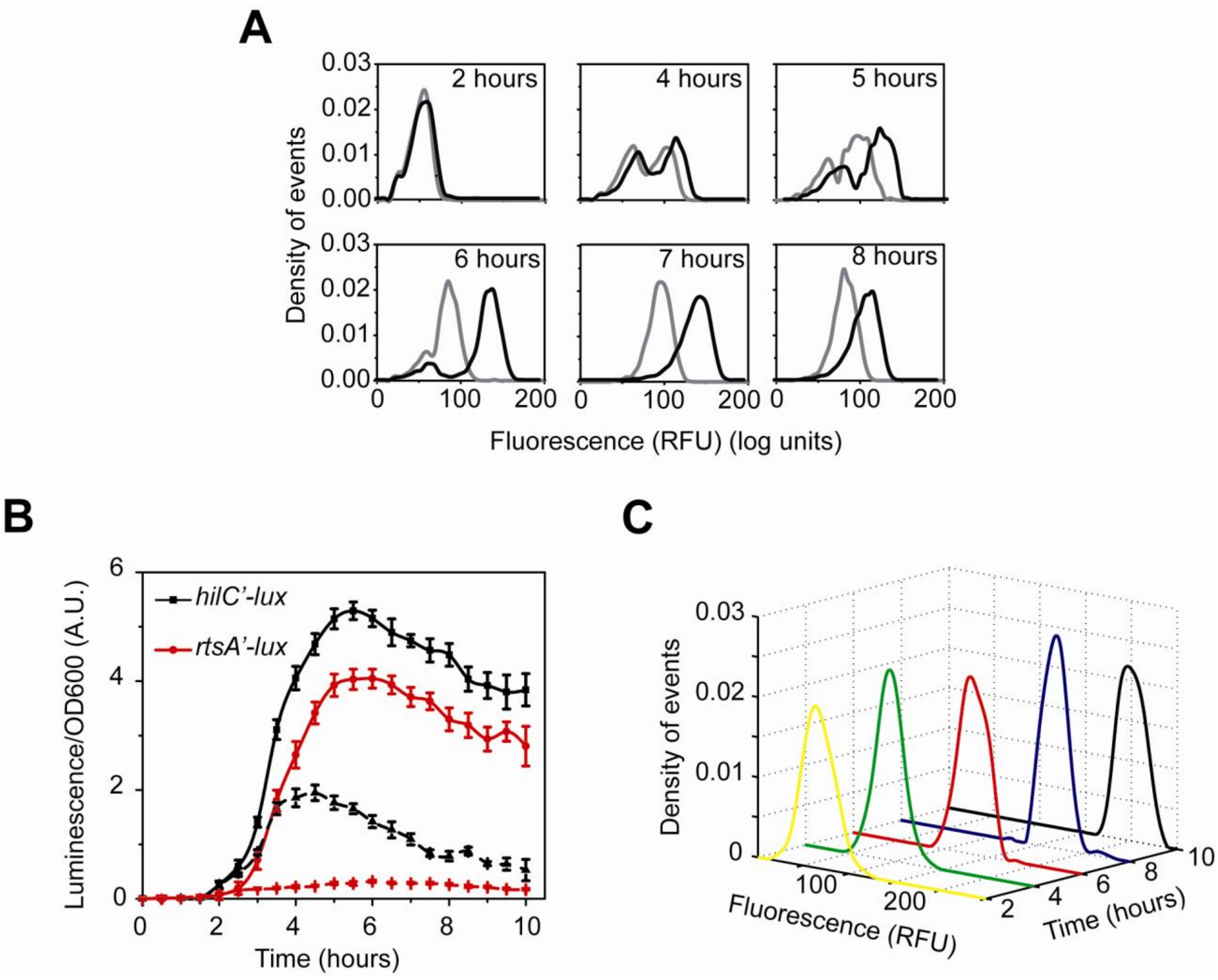

Figure S2. Activation of the SPI1 gene circuit.

(A). Comparison of time-course dynamics of P_{hilD} (pSS072) promoter activities in wild type (black) and a ΔSPI1 Δ*rtsA* mutant (CR349, grey) as determined using green fluorescent protein (GFP) transcriptional fusions and flow cytometry. (B) Comparison of P_{hilC} (pSS075, black) and P_{rtsA} (pSS076, red) promoter activities in wild type (solid lines) and a Δ*hilD* mutant (JS253, dashed lines). Note that the P_{rtsA} promoter is off in the absence of HilD. (C) Dynamics of P_{hilC} (pSS073) promoter activities in a Δ*hilD* mutant (JS253) as determined using green fluorescent protein (GFP) transcriptional fusions and flow cytometry. Note that, in the absence of HilD, the activation of the P_{hilC} promoter is no longer switch-like but instead continuous. Experiments were performed as described in Figure 1.
