# Supplementary material for: The Role of Coupled Positive Feedback in the Expression of the SPI1 Type Three Secretion System in Salmonella
Source: PLoS Pathog. 2010 Jul 29;6(7):e1001025. doi: 10.1371/journal.ppat.1001025 (PMC2912647; doi:10.1371/journal.ppat.1001025)
Supplement: Figure S3 — HilC and RtsA amplify SPI1 gene expression in an additive manner. (A) Normalized PhilD and PhilA promoter activity in wild type (solid) and ΔhilC ΔrtsA (dashed) mutant. The data from Figure 3A were normalized with respect to their maximal value. (B) Comparison of time-course dynamics of PhilA (pSS077) promoter activities in wild type and ΔrtsA (JS248), ΔhilC (JS252), ΔhilC ΔrtsA (CR350), and ΔhilD (CR253) mutants as determined using luciferase transcriptional reporters. (C) Comparison of PhilA (pSS055) promoter activities in wild type (black) and ΔhilC (JS252, red) and ΔrtsA (JS248, grey) mutants as determined using green fluorescent protein (GFP) transcriptional fusions and flow cytometry. (D) Comparison of time-course dynamics of PhilD (pSS074) promoter activities in wild type and ΔrtsA (JS248), ΔhilC (JS252), ΔhilC ΔrtsA (CR350), and ΔhilD (CR253) mutants as determined using luciferase transcriptional reporters. (E) Comparison of PhilD (pSS072) promoter activities in wild type (black) and ΔhilC (JS252, red) and ΔrtsA (JS248, grey) mutants as determined using green fluorescent protein (GFP) transcriptional fusions and flow cytometry. Experiments were performed as described in Figure 1. (0.39 MB PDF) [file ppat.1001025.s003.pdf]

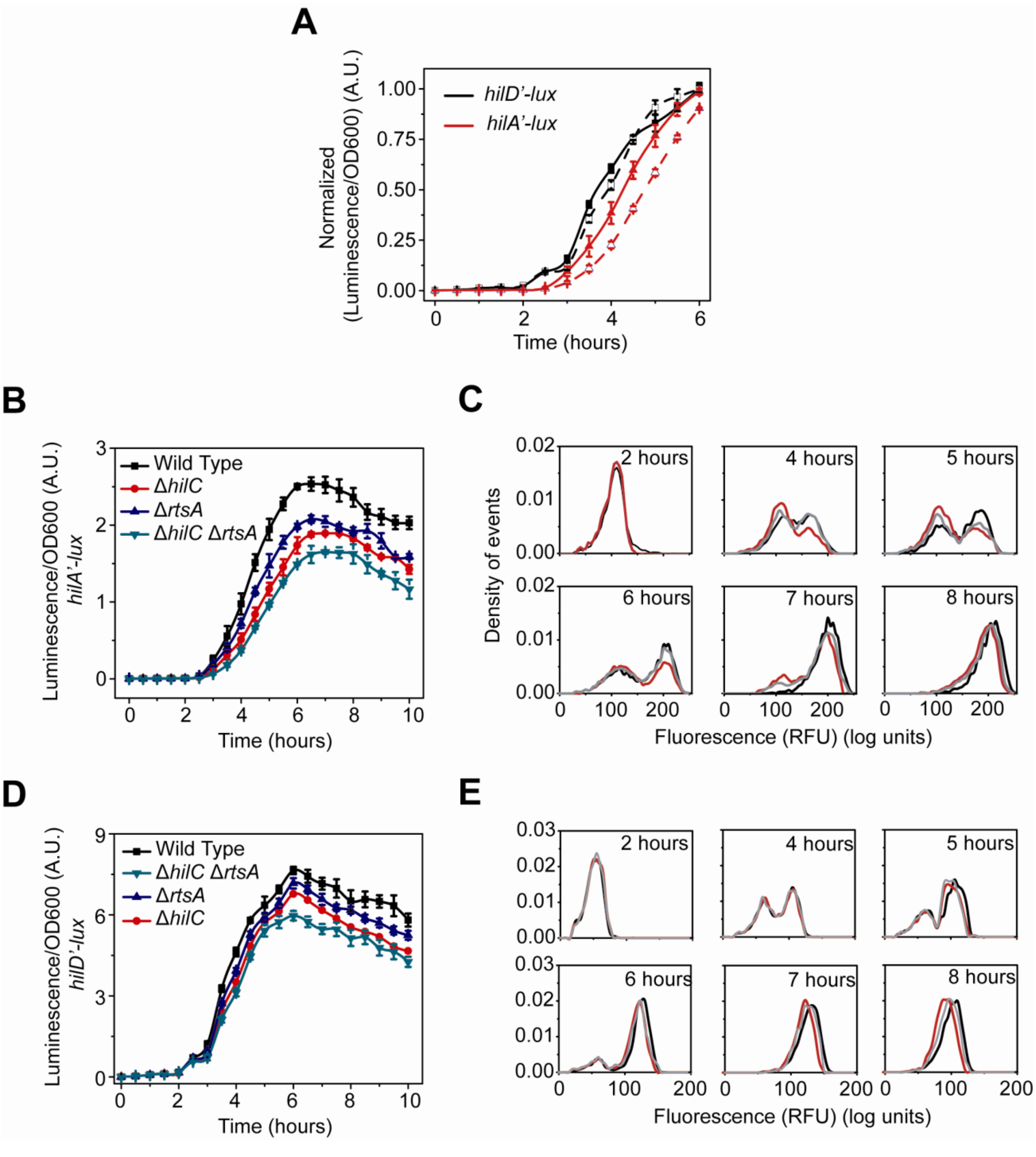

Figure S3. HilC and RtsA amplify SPI1 gene expression in an additive manner.
(A) Normalized P_{hilD} and P_{hilA} promoter activity in wild type (solid) and Δ*hilC* Δ*rtsA* (dashed) mutant. The data from Figure 3A were normalized with respect to their maximal value. (B) Comparison of time-course dynamics of P_{hilA} (pSS077) promoter activities in wild type and Δ*rtsA* (JS248), Δ*hilC* (JS252), Δ*hilC* Δ*rtsA* (CR350), and Δ*hilD* (CR253) mutants as determined using luciferase transcriptional reporters. (C) Comparison of P_{hilA} (pSS055) promoter activities in wild type (black) and Δ*hilC* (JS252, red) and Δ*rtsA* (JS248, grey) mutants as determined using green fluorescent protein

(GFP) transcriptional fusions and flow cytometry. (D) Comparison of time-course dynamics of P_{hilD} (pSS074) promoter activities in wild type and Δ*rtsA* (JS248), Δ*hilC* (JS252), Δ*hilC* Δ*rtsA* (CR350), and Δ*hilD* (CR253) mutants as determined using luciferase transcriptional reporters. (E) Comparison of P_{hilD} (pSS072) promoter activities in wild type (black) and Δ*hilC* (JS252, red) and Δ*rtsA* (JS248, grey) mutants as determined using green fluorescent protein (GFP) transcriptional fusions and flow cytometry. Experiments were performed as described in Figure 1.
