# Supplementary material for: The Role of Coupled Positive Feedback in the Expression of the SPI1 Type Three Secretion System in Salmonella
Source: PLoS Pathog. 2010 Jul 29;6(7):e1001025. doi: 10.1371/journal.ppat.1001025 (PMC2912647; doi:10.1371/journal.ppat.1001025)
Supplement: Figure S4 — HilE negatively regulates HilD expression. (A) Normalized PhilD and PhilA promoter activities in wild type (solid) and ΔhilE (dashed) mutant. The data from Figure 4A was normalized to one for each strain. (B) Comparison of PhilD (pSS072) promoter activities in wild type (black) and ΔhilE (CR361, gray) mutant as determined using green fluorescent protein (GFP) transcriptional fusions and flow cytometry. Experiments were performed as described in Figure 1. (0.12 MB PDF) [file ppat.1001025.s004.pdf]

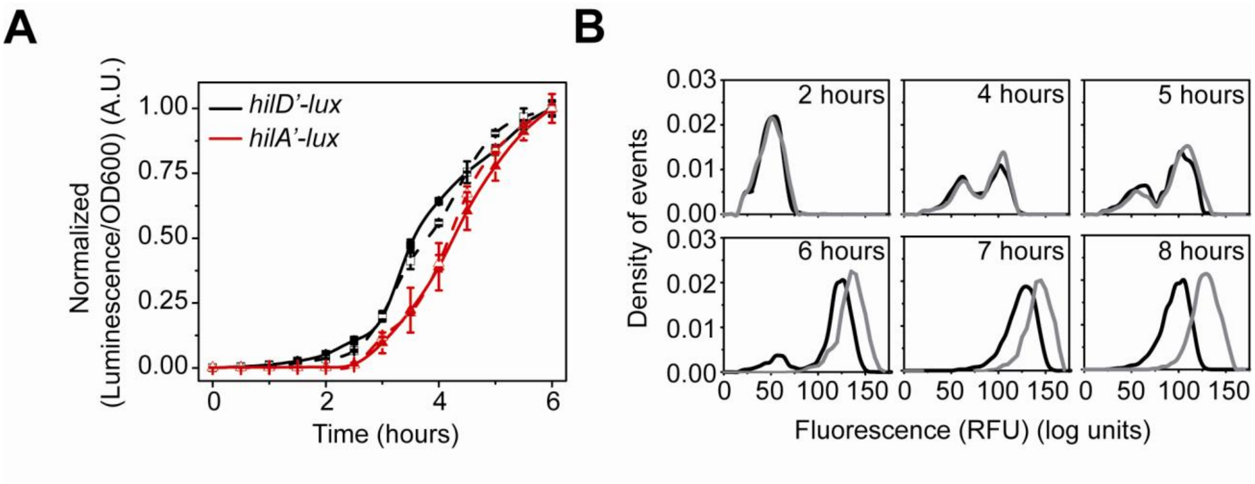

Figure S4. HilE negatively regulates HilD expression.
(A) Normalized P_{hilD} and P_{hilA} promoter activities in wild type (solid) and Δ*hilE* (dashed) mutant. The data from Figure 4A was normalized to one for each strain. (B) Comparison of P_{hilD} (pSS072) promoter activities in wild type (black) and Δ*hilE* (CR361, gray) mutant as determined using green fluorescent protein (GFP) transcriptional fusions and flow cytometry. Experiments were performed as described in Figure 1.
