# Supplementary material for: The Role of Coupled Positive Feedback in the Expression of the SPI1 Type Three Secretion System in Salmonella
Source: PLoS Pathog. 2010 Jul 29;6(7):e1001025. doi: 10.1371/journal.ppat.1001025 (PMC2912647; doi:10.1371/journal.ppat.1001025)
Supplement: Figure S5 — Factors determining SPI1 switch. (A) HilA expression at single-cell resolution when activation of the PhilD promoter is deterministic. In these simulations, we changed the equation for HilD in the model to:All other equations in the model are unchanged. (B) HilA expression at single-cell resolution when the kinetic parameters are reduced by a factor of ten. In our simulations, we accomplished this by reducing λ by a factor of ten and rescaling time by a factor of ten. (C–D) Comparison of HilC expression at single-cell resolution in wild type (C) and ΔhilD mutant (D). Figures are given as two-dimension heat plots, where the color intensity denotes the density of events. The results for each plot were obtained from 1000 simulations. The expression values are normalized to one and plotted on a log scale. The expression values are given in relative log units (R.L.U.). Mutants were simulated by setting the activity of the respective gene to zero in the model. A detailed description of the model is provided in the Materials and Methods. (0.18 MB PDF) [file ppat.1001025.s005.pdf]

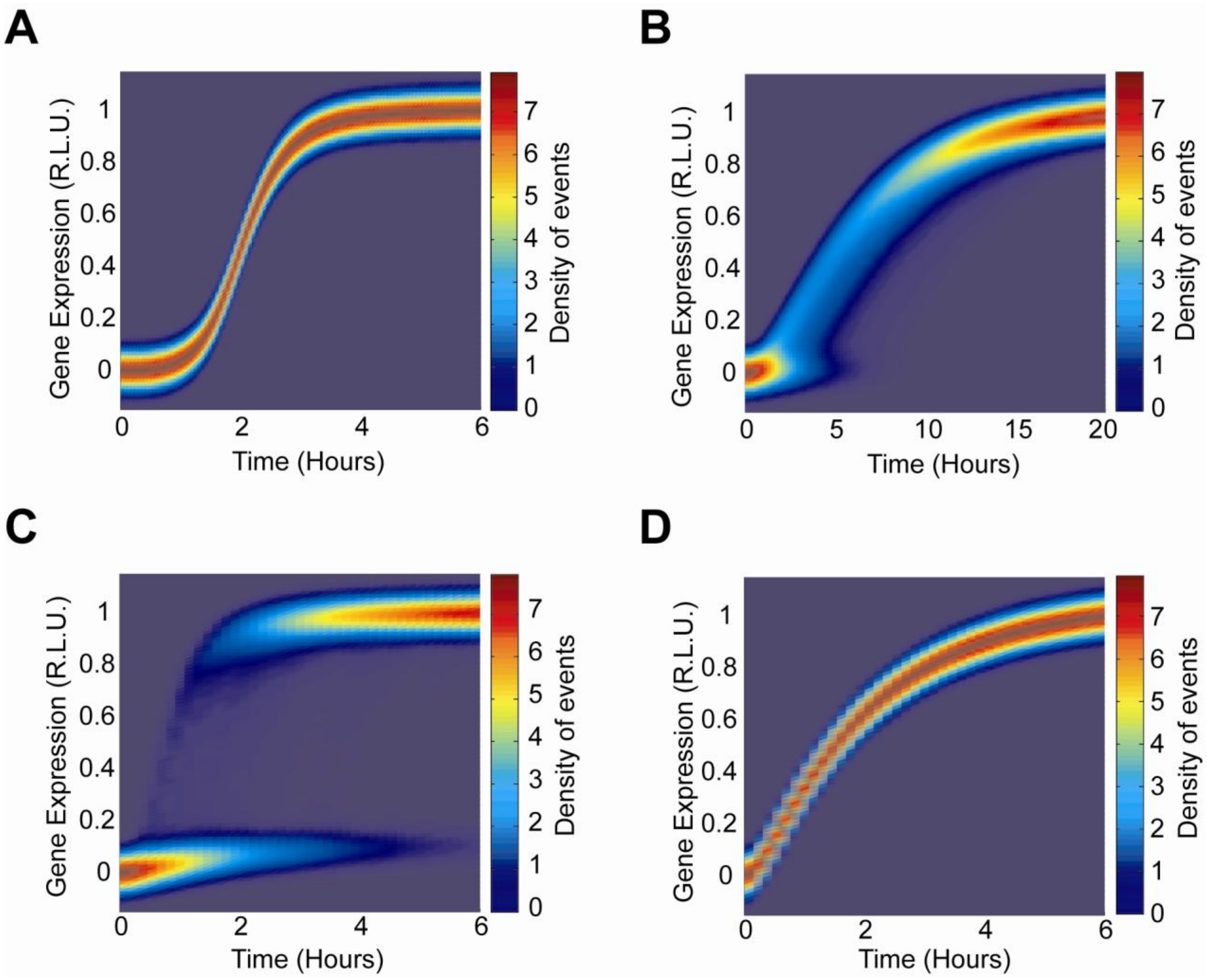

Figure S5. Factors determining SPI1 switch.
(A) HilA expression at single-cell resolution when activation of the P_{hilD} promoter is deterministic. In these simulations, we changed the equation for HilD in the model to:

$$\frac{dD}{dt} = \alpha_D\left(1 - e^{-t/\lambda}\right) + k_D O_1 O_2 - \delta_D D - a_E D \times E + d_E ED.$$

All other equations in the model are unchanged. (B) HilA expression at single-cell resolution when the kinetic parameters are reduced by a factor of ten. In our simulations, we accomplished this by reducing λ by a factor of ten and rescaling time by a factor of ten. (C-D) Comparison of HilC expression at single-cell resolution in wild type (C) and Δ*hilD* mutant (D). Figures are given as two-dimension heat plots, where the color intensity denotes the density of events. The results for each plot were obtained from 1000 simulations. The expression values are normalized to one and plotted on a log scale. The expression values are given in relative log units (R.L.U.). Mutants were simulated by setting the activity of the respective gene to zero in the model. A detailed description of the model is provided in the Materials and Methods.
