# Supplementary material for: The Role of Coupled Positive Feedback in the Expression of the SPI1 Type Three Secretion System in Salmonella
Source: PLoS Pathog. 2010 Jul 29;6(7):e1001025. doi: 10.1371/journal.ppat.1001025 (PMC2912647; doi:10.1371/journal.ppat.1001025)
Supplement: Figure S6 — Parametric analysis of model predicts that SPI1 gene circuit functions as an amplifier and encodes a hard activation threshold. (A) Effect of HilD positive feedback on HilA expression. Plot shows steady-state concentration of HilD as a function of the parameters kD and αD. The parameter kD specifies the degree by which the SPI1 regulators - HilC, HilD, and RtsA - can activate HilD expression, effectively the strength of positive feedback on HilD expression. The parameter αD specifies the strength of the signal activating HilD expression. (B) Effect of HilC and RtsA on HilA expression. Plot shows the steady-state concentration of HilD as a function of the parameters kC, kR, and αD. The parameters kC and kR specify the degree by which the SPI1 regulators - HilC, HilD, and RtsA - can activate HilC and RtsA expression, respectively. In other words, these parameters set the strength of feedback on HilC and RtsA expression. In these simulations, the parameters kC and kR were both varied in tandem: the numerical values for the two are the same. (C) Effect of HilE on HilA expression. Plot shows the steady-state concentration of HilD as a function of the parameters αE and αD. (D–E) Effect of HilE and HilD positive feedback on HilD (D) and HilA (E) expression. Plots shows the steady-state concentrations of HilD and HilA as a function of the parameters αE and kD. The black lines in the plots (A–C) are used to denote the results obtained using the nominal parameters (aside from αE). A detailed description of the model is provided in the Materials and Methods. (0.79 MB PDF) [file ppat.1001025.s006.pdf]

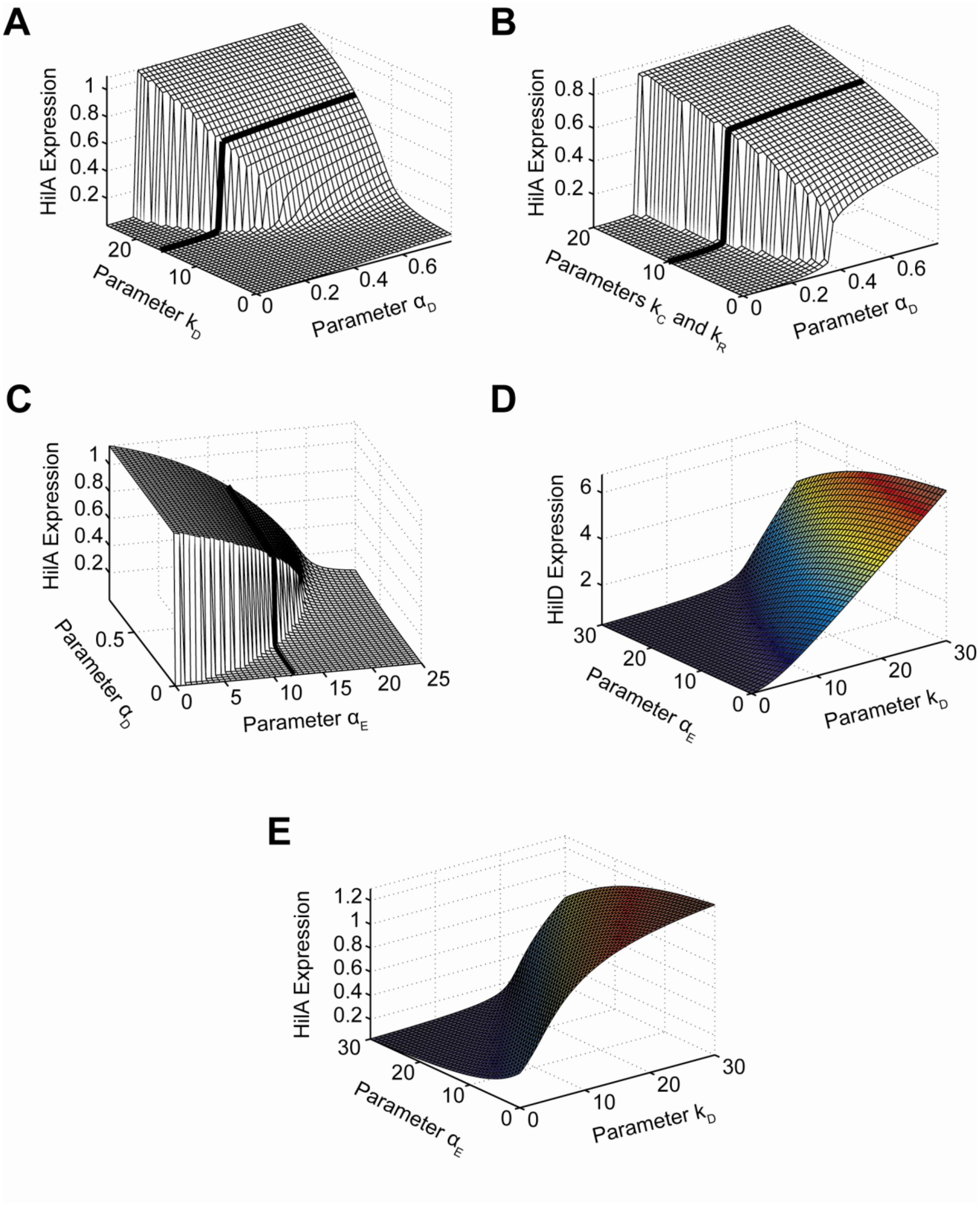

Figure S6. Parametric analysis of model predicts that SPI1 gene circuit functions as an amplifier and encodes a hard activation threshold.

(A) Effect of HilD positive feedback on HilA expression. Plot shows steady-state concentration of HilD as a function of the parameters k_D and α_D. The parameter k_D

specifies the degree by which the SPI1 regulators - HilC, HilD, and RtsA - can activate HilD expression, effectively the strength of positive feedback on HilD expression. The parameter α_D specifies the strength of the signal activating HilD expression. (B) Effect of HilC and RtsA on HilA expression. Plot shows the steady-state concentration of HilD as a function of the parameters k_C, k_R, and α_D. The parameters k_C and k_R specify the degree by which the SPI1 regulators - HilC, HilD, and RtsA - can activate HilC and RtsA expression, respectively. In other words, these parameters set the strength of feedback on HilC and RtsA expression. In these simulations, the parameters k_C and k_R were both varied in tandem: the numerical values for the two are the same. (C) Effect of HilE on HilA expression. Plot shows the steady-state concentration of HilD as a function of the parameters α_E and α_D. (D-E) Effect of HilE and HilD positive feedback on HilD (D) and HilA (E) expression. Plots shows the steady-state concentrations of HilD and HilA as a function of the parameters α_E and k_D. The black lines in the plots (A-C) are used to denote the results obtained using the nominal parameters (aside from α_E). A detailed description of the model is provided in the Materials and Methods.
