# Supplementary material for: The Role of Coupled Positive Feedback in the Expression of the SPI1 Type Three Secretion System in Salmonella
Source: PLoS Pathog. 2010 Jul 29;6(7):e1001025. doi: 10.1371/journal.ppat.1001025 (PMC2912647; doi:10.1371/journal.ppat.1001025)
Supplement: Figure S7 — SPI1 promoter activities are linear correlated to one another at varying levels of induction and in different mutants. (A) Correlation of PhilD (pSS052) promoter activity with PhilA (pSS055), PhilC (pSS053), and PrtsA (pSS054) promoter activities. To induce SPI1 gene expression, cells were first grown overnight in LB/no salt and then sub-cultured into fresh LB at varying concentrations of NaCl to an OD of 0.05 and grown statically for 15 hours. Individual experiments used to construct correlations are given in Panels B–E. (B–E) Comparison of PhilD (pSS052, B), PhilC (pSS053, C), PhilA (pSS055, D), and PrtsA (pSS054, E) promoter activities at varying concentration of NaCl and in different mutant backgrounds as determined using GFP transcriptional reporters. Fluorescence values were normalized with the OD600 absorbance to account for cell density. Data is the average of three independent experiments. Error-bars denote standard deviation. (0.36 MB PDF) [file ppat.1001025.s007.pdf]

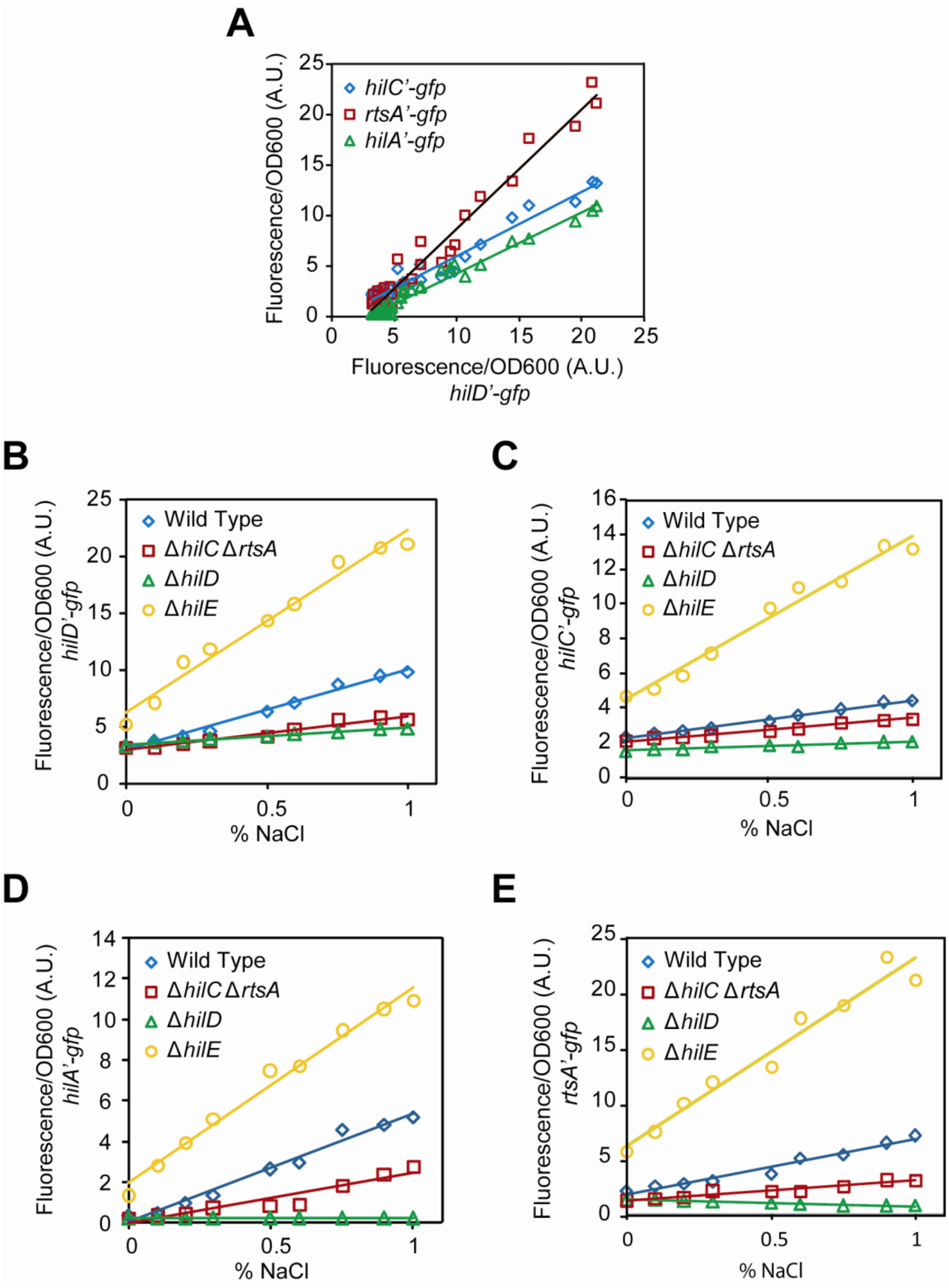

Figure S7. SPI1 promoter activities are linear correlated to one another at varying levels of induction and in different mutants.

(A) Correlation of P_{hilD} (pSS052) promoter activity with P_{hilA} (pSS055), P_{hilC} (pSS053), and P_{rtsA} (pSS054) promoter activities. To induce SPI1 gene expression, cells were first

grown overnight in LB/no salt and then sub-cultured into fresh LB at varying concentrations of NaCl to an OD of 0.05 and grown statically for 15 hours. Individual experiments used to construct correlations are given in Panels B-E. (B-E) Comparison of P_{hilD} (pSS052, B), P_{hilC} (pSS053, C), P_{hilA} (pSS055, D), and P_{rtsA} (pSS054, E) promoter activities at varying concentration of NaCl and in different mutant backgrounds as determined using GFP transcriptional reporters. Fluorescence values were normalized with the OD600 absorbance to account for cell density. Data is the average of three independent experiments. Error-bars denote standard deviation.
